# Rigid‐Flexible Coupling Units Enable Robust Large Birefringence

**DOI:** 10.1002/advs.74948

**Published:** 2026-03-26

**Authors:** Jie Zhou, Yanqiang Li, Ji Qi, Weiqi Huang, Liangmeng Zhu, Zhiyong Bai, Yang Zhou, Junhua Luo, Sangen Zhao

**Affiliations:** ^1^ State Key Laboratory of Functional Crystals and Devices Fujian Institute of Research on the Structure of Matter Chinese Academy of Sciences Fuzhou Fujian China; ^2^ College of Chemistry Fuzhou University Fuzhou Fujian China; ^3^ Fujian College University of Chinese Academy of Sciences Fuzhou Fujian China; ^4^ Quantum Science Center of Guangdong‐Hong Kong‐Macao Greater Bay Area Shenzhen Guangdong China

## Abstract

Birefringent crystals are central to polarization control in laser technologies (e.g., wave plates and polarization beam splitters), yet combining large birefringence with robust optical stability remains rare. Herein, we demonstrate the rigid–flexible coupling strategy exemplified by a novel crystal, Sr_4_(VO_4_)_2_S_3_, constructed from rigid (VO_4_)^3−^ tetrahedra and flexible linear (S_3_)^2−^ units. Sr_4_(VO_4_)_2_S_3_ exhibits an exceptional birefringence (Δ*n* = 0.52@550 nm), exceeding commercial YVO_4_ by 249%. Remarkably, the birefringence remains robust over a broad temperature range (123–373 K) and after prolonged exposure to air and water. Structural analyses reveal that Sr–O/S polyhedra are interconnected with rigid (VO_4_)^3−^ units to build the overall 3D framework that underpins robustness, which also facilitates the optimal alignment of flexible (S_3_)^2−^ units. Theoretical calculations further indicate that (S_3_)^2−^ units possess much higher bond flexibility than (VO_4_)^3−^ tetrahedra (flexible index of S–S 4.33 vs. V–O 0.28), thereby governing the prominent optical birefringence. This work establishes rigid–flexible coupling as a general design principle to unite large birefringence with robustness for compact, reliable polarization optics.

## Graphical abstract

A rigid–flexible coupling strategy, combining flexible (S_3_)^2−^ linear units with rigid (VO_4_)^3−^ tetrahedra, yields Sr_4_(VO_4_)_2_S_3_—a robust crystal exhibiting large birefringence (Δ*n* = 0.52@550 nm) and remarkable thermal, air, and water stability.

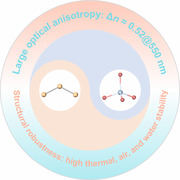

## 1 Introduction

When light propagates through an optically anisotropic crystal, it splits into two distinct rays, which are orthogonally polarized and travel at different speeds, namely the ordinary and extraordinary rays [1, 2]. This phenomenon enables birefringent crystals to modulate polarized light, making them indispensable in laser technologies, such as polarizers, wave plates, and polarization beam splitters [3, 4, 5, 6, 7]. Large birefringence is particularly desirable, as it allows a target phase retardation or polarization separation to be achieved with a thinner crystal. This not only facilitates more compact devices but also alleviates the need for large‐sized single crystals [4, 8]. Furthermore, birefringent crystals must also possess robust optical performance to meet the increased demand for high laser power density and long‐term operational stability [9, 10, 11]. However, most commercial birefringent crystals exhibit relatively modest birefringence below 0.3, such as MgF_2_ (Δ*n*_exp_ = 0.011@532 nm) [12], LiNbO_3_ (Δ*n*_exp_ = 0.074@546 nm) [13], *α*‐BaB_2_O_4_ (Δ*n*_exp_ = 0.122@532 nm) [14], CaCO_3_ (Δ*n*_exp_ = 0.172@532 nm) [15], YVO_4_ (Δ*n*_exp_ = 0.209@532 nm) [16]. These limitations motivate the exploration of new birefringent crystals that combine robustness with large birefringence.

**SCHEME 1:**
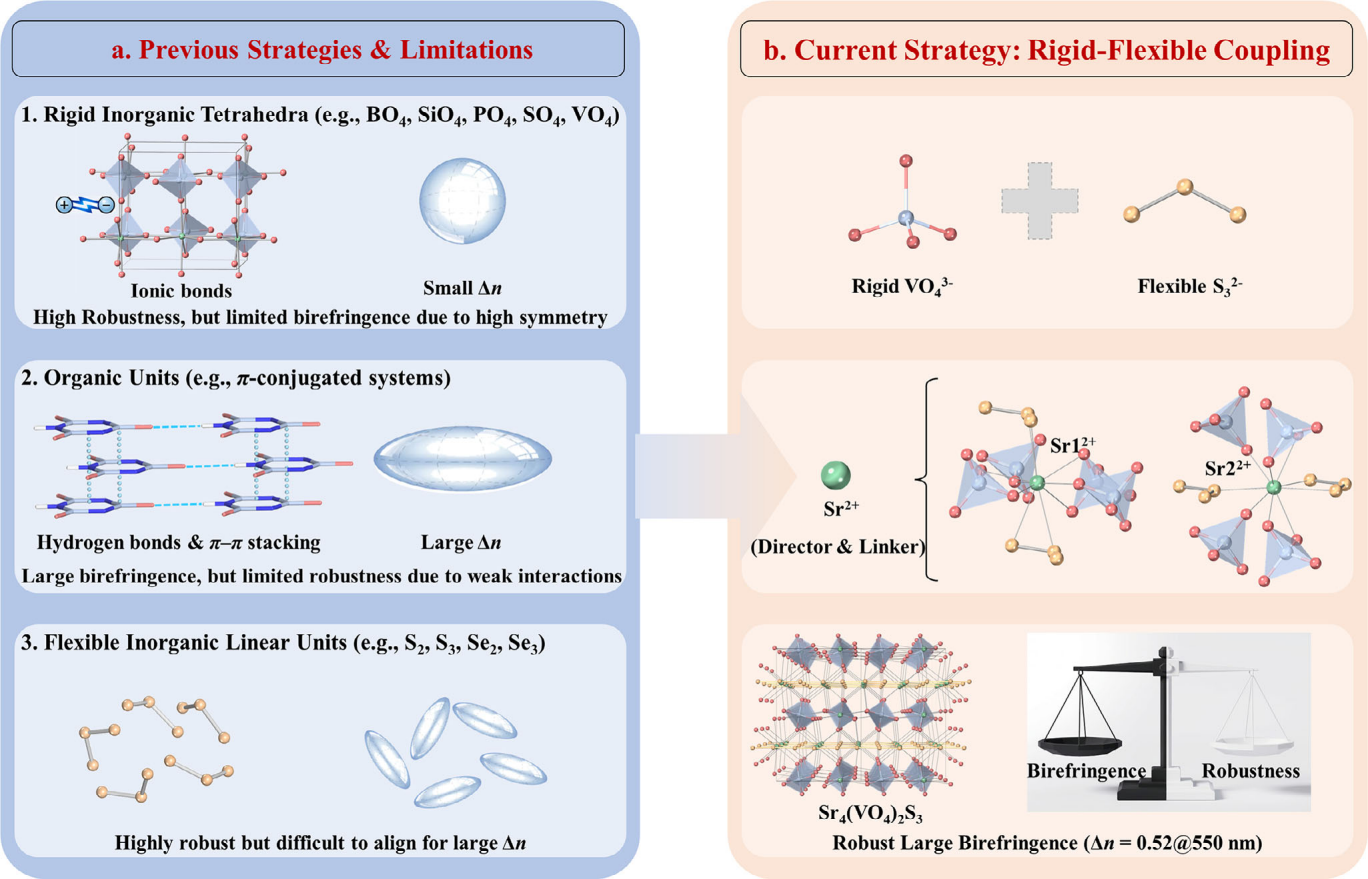
The strategy for constructing a robust large birefringence. (a) Previous design strategies for birefringent crystals and their limitations. (b) Our strategy for achieving robust large birefringence by rigid–flexible coupling.

Despite their structural rigidity, inorganic tetrahedral oxyanions (e.g., (SO_4_)^2−^, (PO_4_)^3−^, (SiO_4_)^4−^, (BO_4_)^5−^, and (VO_4_)^3−^) are well known to exhibit intrinsically limited optical anisotropy due to near‐*T_d_* symmetry (Scheme 1a‐1) [17, 18, 19, 20]. In contrast, organic planar *π*‐conjugated units display pronounced in‐plane and out‐of‐plane polarizability differences arising from the *π*‐electron delocalization, enabling numerous hybrid birefringent crystals with large birefringence, including (C_2_N_4_OH_7_)(NH_2_SO_3_) (Δ*n*_cal_ = 0.225@1064 nm) [21], (4‐HPy)(CH_3_SO_3_) (Δ*n*_exp_ = 0.216@546 nm) [22], (4‐HPy)(H_2_PO_4_) (Δ*n*_cal_ = 0.25@1064 nm) [23], (2‐AP)(NH_2_SO_3_) (Δ*n*_exp_ = 0.233@546 nm) [24], and K(3‐PySO_3_) (Δ*n*_exp_ = 0.306@546 nm) [25], Unfortunately, these hybrids are typically assembled through weak intermolecular interactions like hydrogen bonding and *π*–*π* stacking, which often compromise robustness (thermal, water, and even air stability), thereby severely hindering their practical applications (Scheme 1a‐2) [21, 22, 23, 24, 25, 26, 27, 28].

Recently, flexible inorganic linear units with deformable electron clouds have emerged as promising candidates for birefringent building blocks, such as (BO_2_)^−^, (S_2_)^2−^, and (S_3_)^2−^ [11, 29, 30, 31]. For example, by integrating (S_2_)^2−^ and (S_3_)^2−^, Pan and co‐workers reported an infrared birefringent crystal Na_4_Ba_3_(S_2_)_4_S_3_, which shows robust birefringence of Δ*n*_cal_ = 0.37@1064 nm. Nevertheless, it remains challenging to realize an optimal arrangement of such flexible units within the crystal lattice, which is crucial for amplifying macroscopic birefringence (Scheme 1a‐3).

In this work, we proposed a rigid–flexible coupling strategy, in which rigid units serve as a robust structural scaffold, while flexible linear units act as the dominant microscopic sources of birefringence. Crucially, charge‐balancing cation‐centered coordination polyhedra optimize the spacing of flexible units, linking them to rigid units to construct a robust 3D framework (Scheme 1b). Guided by this principle, we coupled flexible linear (S_3_)^2−^ and rigid tetrahedral (VO_4_)^3−^, and reported a novel vanadate, namely Sr_4_(VO_4_)_2_S_3_ (**I**). In the crystal structure, Sr–O/S polyhedra are linked with rigid (VO_4_)^3−^ tetrahedra via sharing corners to construct a robust 3D framework, while directing the parallel arrangement of flexible (S_3_)^2−^ units. Compound **I** exhibits the giant birefringence of Δ*n* = 0.52@550 nm and high thermal stability up to 957 K, as well as outstanding air and water stability. Furthermore, its birefringence remains robust over a wide temperature range (123–373 K) and after exposure to air and water for 7 days. This exceptional combination of robustness and large birefringence outperforms that of current commercial birefringent crystals.

## 2 Results and Discussion

**FIGURE 1:**
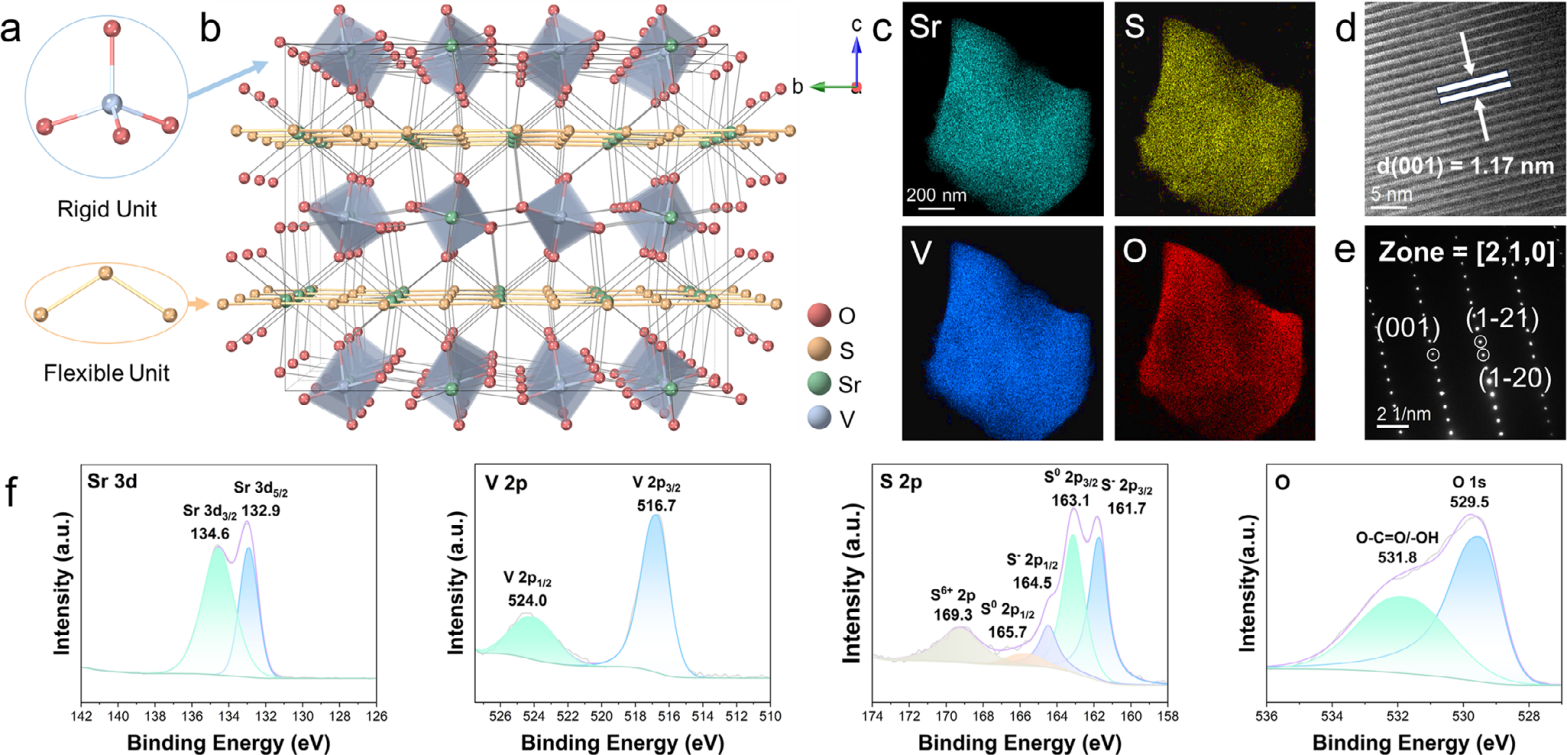
Crystal structure and chemical composition of **I**. (a) Rigid tetrahedral (VO_4_)^3−^ and flexible linear (S_3_)^2−^. (b) Crystal structure of **I** along the *a* axis. (c) EDS elemental mapping of a single crystal of **I**. (d) HRTEM image and (e) SAED pattern of **I**. (f) XPS fine spectra for Sr 3d, V 2p, S 2p, and O 1s.

Compound **I** was synthesized by the high‐temperature solid‐state reaction, with detailed synthetic procedures provided in the Supporting Information. Based on the single‐crystal X‐ray diffraction (XRD), we determined the crystal structure of **I**, which crystallizes in the orthorhombic space group *Pbcm* (No. 57) with unit cell parameters *a* = 6.6556 Å, *b* = 7.4628 Å, *c* = 11.6900 Å, and *Z* = 2 (Table S1–S5). The asymmetric unit comprises two crystallographically independent Sr^2+^ cations (Sr1 and Sr2), one (VO_4_)^3−^ tetrahedron, and one linear (S_3_)^2−^ group with 50% occupancy. Sr2 is nine‐coordinated by six O atoms and three S atoms, forming a Sr2O_6_S_3_ polyhedron (Figure S1a), whereas Sr1 adopts a slightly higher coordination environment of Sr1O_6_S_4_ (Figure S1b). These Sr‐centered polyhedra are interconnected via sharing edges and faces, which are further bridged by rigid tetrahedral (VO_4_)^3−^ (Figure 1a) to construct the overall robust 3D framework (Figure 1b). In addition, they act as geometrically constrained structural directors: the directionally oriented Sr–S coordination sites on the polyhedral walls favor optimal arrangement of the flexible (S_3_)^2−^ units (Figure 1a). Within each (S_3_)^2−^ unit, S─S covalent bond lengths are 2.065(3) Å and 2.064(3) Å, consistent with values reported for other polysulfides [32]. As shown in Figure 1b and Figure S2, the partially occupied (S_3_)^2−^ units are constrained by the *b*‐glide plane at (0, *y*, *z*) and adopt alternating orientations, forming quasi‐1D [S_3_]_∞_ chains along the *b*‐axis. Adjacent [S_3_]_∞_ chains are further stacked in an antiparallel manner, resulting in an overall centrosymmetric crystal structure. It should be noted that this arrangement induces pronounced structural anisotropy along the *b*‐axis relative to the *a*‐ and *c*‐axes, suggesting the potential for giant optical anisotropy in **I**. The distortion of the (VO_4_)^3−^ tetrahedra was quantified by the parameter $\Delta d=\frac{1}{4}\;\Sigma_{i\;=1}^{4}{\left[\frac{d_{i}-d}{d}\right]}^{2}$, where *d_i_* represents an individual V─O bond length and *d* is their average [33]. The calculated Δ*d* value of 3.80 × 10^−5^ is significantly larger than that of the famous vanadate YVO_4_ (Δ*d* = 0) [34], confirming evident distortion in the (VO_4_)^3−^ tetrahedra of **I**, which further promotes the anisotropic optical responses. The V─O (1.701(1)–1.722(1) Å), Sr─O (2.510(1)–2.793(1) Å), and Sr─S (2.949(3)–3.299(3) Å) distances are all reasonable according to previous reports [17, 32, 34]. Bond valence sum (BVS) calculations were performed for each constituent atom to further validate the structural model. The obtained BVS values are: Sr1, +2.253; Sr2, +2.146; V1, +5.128; S2A, −1.032; S2B, −1.065; O1, −2.057; O2, −2.041, which agree well with the expected states (Sr^2+^, V^5+^, S^−^, and O^2−^).

The powder XRD pattern of the polycrystalline samples of compound **I** was well indexed and showed excellent agreement with the simulated pattern based on single‐crystal structural data, confirming the phase purity of **I** (Figure S3). The powder patterns remained unchanged after four months of exposure to air, indicating excellent air stability of **I** (Figure S3). In addition, there was little change in the surface morphology of the selected **I** crystal after being immersed in water for one week (Figure S4), demonstrating the outstanding water stability of **I**. The thermal stability of **I** was investigated by thermogravimetry and differential thermal analyses, from which **I** remains thermally stable up to 957 K (Figure S5), a temperature notably lower than that required for crystal growth. This discrepancy likely arises because Sr_4_(VO_4_)_2_S_3_ single crystals were grown in a sealed tube under a sulfur‐rich self‐flux environment, where the sulfur amount used significantly exceeds the stoichiometric requirement. By contrast, the reported thermal stability limit was measured in a flowing N_2_ with low sulfur chemical potential, which promotes sulfur loss and thus earlier decomposition. Consistently, powder XRD after heating under flowing N_2_ agrees with the simulated pattern at 940 K but deviates significantly at 980 K (Figure S6), confirming decomposition above 957 K under inert‐gas thermogravimetry conditions. Similar behavior has also been observed for related vanadium oxysulfides (e.g., Ba_5_(VO_2_S_2_)_2_(S_2_)_2_) [35]. As demonstrated in Figure 1c and Figure S7, energy‐dispersive X‐ray spectroscopy (EDS) mapping confirmed the expected homogeneous distribution of Sr, S, V, and O elements throughout the tested crystal. High‐resolution transmission electron microscopy (HRTEM) reveals an interplanar spacing of 1.17 nm, corresponding to the (001) plane (Figure 1d). Moreover, the sharp diffraction spots in the selected‐area electron diffraction (SAED) pattern (Figure 1e) further verify the high crystallinity of the as‐synthesized **I** samples.

X‐ray photoelectron spectroscopy (XPS) was further employed to investigate the chemical composition of **I** (Figure 1f), in which Sr 3d, V 2p, S 2p, and O 1s signals were clearly observed. The fine XPS spectrum of Sr 3d exhibits two peaks at approximately 132.9 and 134.6 eV, corresponding to Sr 3d_5/2_ and Sr 3d_3/2_, respectively [36]. In the fine spectrum of V 2p, there are two major peaks at about 516.7 and 524.0 eV, coming from V 2p_3/2_ and V 2p_1/2_ [37]. As for the S 2p spectrum, the peaks at 164.5 and 161.7 eV belong to 2p_1/2_ and 2p_3/2_ of S^−^. The other peaks at 165.7 and 163.1 eV derive from 2p_1/2_ and 2p_3/2_ of S^0^ of (S_3_)^2−^ [38, 39]. The fine spectrum of O 1s contains two peaks at 529.5 and 531.8 eV, in which the strongest peak at 529.5 eV is related to V─O [37]. The other weaker peak located at 531.8 eV may be associated with adsorbed H_2_O and CO_2_ [40, 41].

Raman spectroscopy was presented in Figure S8, where characteristic vibrational modes of the (S_3_)^2−^ and (VO_4_)^3−^ can be clearly observed. The strong peak located at 472.2 cm^−1^ corresponds to the stretch vibration of (S_3_)^2−^, while the lower frequency peak at 252.9 cm^−1^ is attributed to its bending mode [42, 43]. In addition, some scattering peaks observed between 750 and 900 cm^−1^ are assigned to the V─O stretching modes [44]. As shown in Figure S9, these vibrational features are also evident in the Fourier transform infrared spectroscopy (FTIR). Notably, no significant absorption bands were observed in the range of 4000–1111 cm^−1^, indicating that **I** possesses an infrared transparency window of approximately 9.0 µm. Moreover, the UV–vis–NIR diffuse reflectance spectroscopy of **I** was collected on a Lambda 950 UV–vis–NIR spectrophotometer. Based on the Kubelka‐Munk function, the optical bandgap of **I** was calculated to be 2.3 eV (Figure S10). Taken together with the UV–vis–NIR and FTIR results, **I** exhibits a relatively wide transparency window ranging from 490 to 9000 nm.

To investigate the optical anisotropy of **I**, polarized Raman spectroscopy (PRS) of **I** was collected at *λ* = 532 nm [45]. A half‐wave plate was placed in the path of the incident beam, allowing rotation of the polarization directions of the incident beam. An analyzer was placed in the scattered‐beam path to select parallel or perpendicular configurations. The intensity of Raman signals was proportional to:*I*∝|*e_i_* × *R* × *e_s_* |^2^ [46, 47], where *e_i_* and *e_s_* represent the polarization unit vectors of the incident and scattered light, respectively. Specifically, the incident polarization vector lies in the (001) crystal plane and is defined as *e_i_* =  (cos θ, sin θ,  0), with 𝜃 denoting the angle between the polarization direction of the incident light and the crystallographic axis. In the parallel polarization setup, *e_s_* =  (1,  0,  0), whereas in the perpendicular polarization setup, *e_s_* = (0, 1, 0) . Given that compound **I** crystallizes in the orthorhombic space group *Pbcm* (No. 57), the A_g_, B_1g_, B_2g_, and B_3g_ vibrational modes are Raman‐active. The corresponding Raman tensors are as follows [46]:

$$R\;\left(A_{g}\right)=\left[\begin{array}{ccc} a & 0 & 0\\ 0 & b & 0\\ 0 & 0 & c \end{array}\right]$$

$$R\;\left(B_{1g}\right)=\left[\begin{array}{ccc} 0 & d & 0\\ d & 0 & 0\\ 0 & 0 & 0 \end{array}\right]$$

$$R\;\left(B_{2g}\right)=\left[\begin{array}{ccc} 0 & 0 & e\\ 0 & 0 & 0\\ e & 0 & 0 \end{array}\right]$$

$$R\;\left(B_{3g}\right)=\left[\begin{array}{ccc} 0 & 0 & 0\\ 0 & 0 & f\\ 0 & f & 0 \end{array}\right]$$

Accordingly, the angular dependent Raman scattering intensity in both parallel (∥) and perpendicular (⊥) polarization configurations can be expressed as:

$$I\left(\mathrm{Ag},∥\right)\propto a^{2}\cos^{2}\theta$$

$$I\left(B_{1g},∥\right)\propto d^{2}\sin^{2}\theta$$

$$I\;\left(B_{2g},∥\right)=I\;\left(B_{3g},∥\right)=0$$

$$I\left(\mathrm{Ag},⊥\right)\propto b^{2}\sin^{2}\theta$$

$$I\left(B_{1g},⊥\right)\propto d^{2}\cos^{2}\theta$$

$$I\left(B_{2g},⊥\right)=I\left(B_{3g},⊥\right)=0$$

**FIGURE 2:**
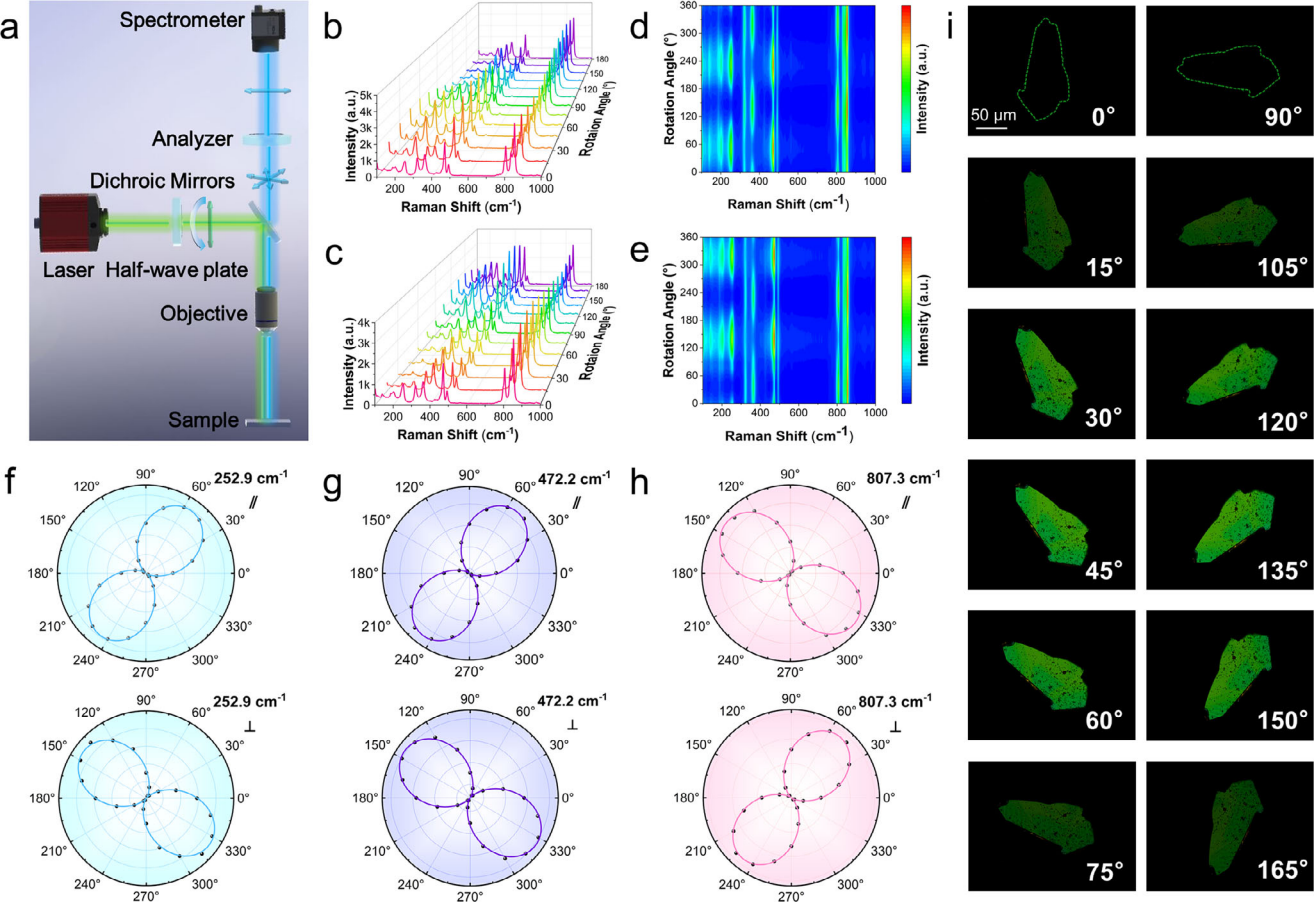
Anisotropic characterization for **I**. (a) Schematic diagram of PRS measurement. 3D PRS maps under (b) parallel‐ and (c) perpendicular‐polarization configurations. Corresponding 2D contour plots under (d) parallel and (e) perpendicular configurations. Polar plots of polarized Raman intensity under 252.9 cm^−1^, (f) 472.2 cm^−1^ (g), and 807.3 cm^−1^ (h). (i) Cross‐polarized optical images recorded in increments of 15° from 0° to 165°.

Figure 2b–e displays the 3D PRS maps and corresponding 2D contour maps of **I** under parallel and perpendicular polarization configurations, respectively, with the half‐wave plate rotated from 0° to 360° in 15° increments. There was a periodic variation in Raman intensity that indicates anisotropic molecular vibrations in **I**. The polar plots of the fitted peak intensities as a function of sample rotation angle at characteristic wavenumbers of 252.9, 472.2, and 807.3 cm^−1^ were shown in Figure 2f–h. Under both polarization configurations, the peaks show a distinct two‐lobed shape, oriented perpendicularly to each other, with a periodicity of 180°. These results are in good agreement with the theoretical predictions and confirm the optical anisotropy in **I**. We further conducted angle‐dependent optical observations to reveal the optical anisotropy of **I**. As shown in Figure 2i, at *α* = 0°, no light is transmitted to the analyzer, indicating complete extinction. With increasing *α* from 0° to 45°, the transmitted light intensity gradually increases, resulting in a brighter interference color. At *α* = 45°, interference color reaches maximum brightness. In other words, as the tested crystal rotates, the brightness of the interference color varies periodic variation every 45° (Figure 2i). These findings unambiguously demonstrate the pronounced optical anisotropy of **I**, i.e., its birefringence.

**FIGURE 3:**
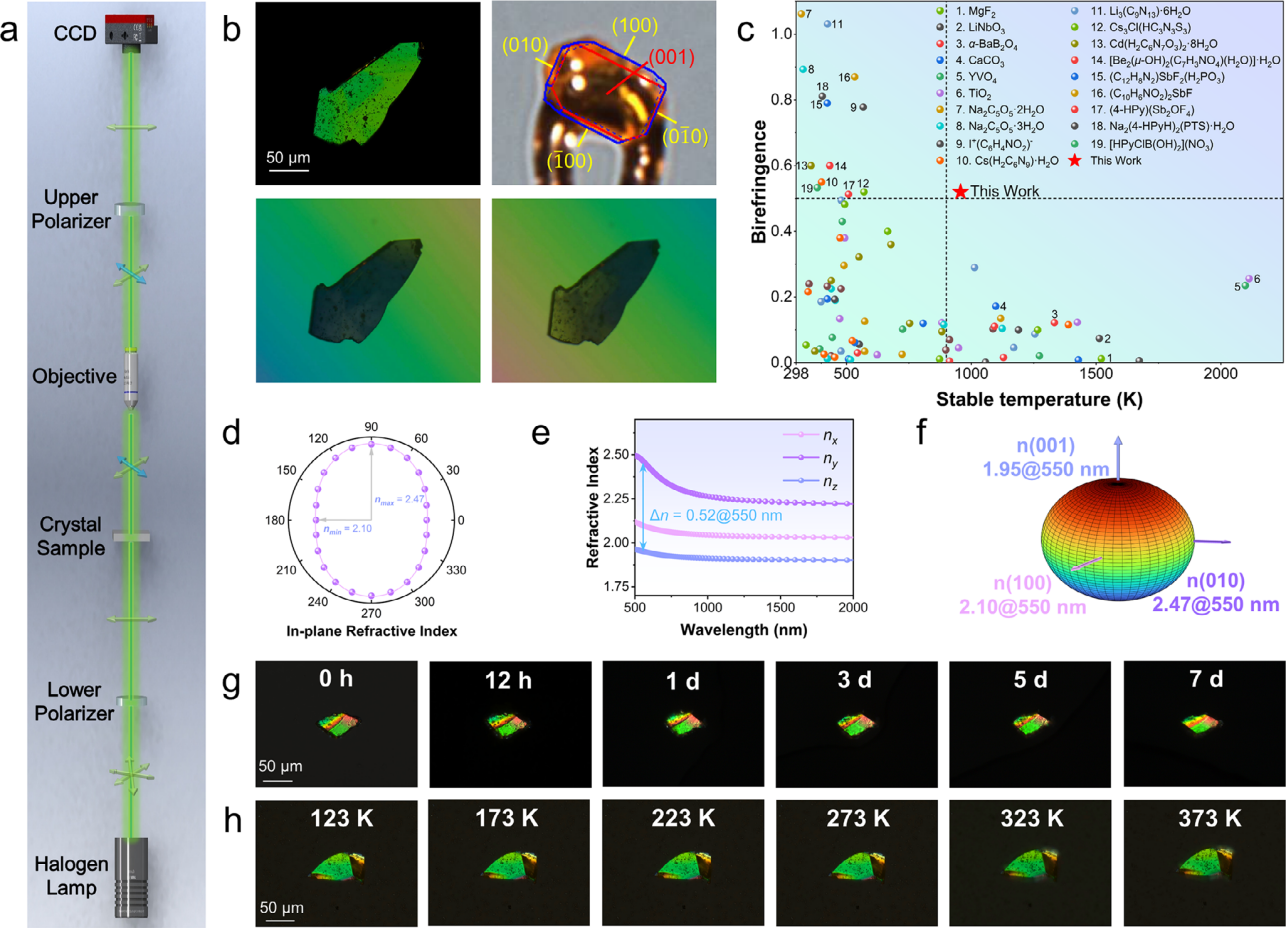
Birefringence properties of **I**. (a) Schematic diagram of the polarized microscope method. (b) Top row: from left to right, original interference color of the crystal sample under the orthogonally polarized light of **I**. Crystal orientation observed via the single‐crystal XRD (inset: the tested single crystal attached to a nylon loop). Bottom row: complete extinction of the **I** crystal by rotating the compensator either clockwise or counterclockwise. (c) Comparison of the birefringence and stable temperature of **I** with those of all commercial birefringent crystals, recently reported famous birefringent crystals, and optical crystals composed of tetrahedral structural units. Commercial birefringent crystals and recently reported giant birefringent crystals with Δ*n* > 0.5 are marked in Figure 3c. (1. MgF_2_, 2. LiNbO_3_, 3. *α*‐BaB_2_O_4_, 4. CaCO_3_, 5. YVO_4_, 6. TiO_2_, 7. Na_2_C_5_O_5_·2H_2_O, 8. Na_2_C_5_O_5_·3H_2_O, 9. I^+^(C_6_H_4_NO_2_)^−^, 10. Cs(H_2_C_6_N_9_)·H_2_O, 11. Li_3_(C_9_N_13_)·6H_2_O, 12. Cs_3_Cl(HC_3_N_3_S_3_), 13. Cd(H_2_C_6_N_7_O_3_)_2_·8H_2_O, 14. [Be_2_(*µ*‐OH)_2_(C_7_H_3_NO_4_)(H_2_O)]·H_2_O, 15. (C_12_H_8_N_2_)SbF_2_(H_2_PO_3_), 16. (C_10_H_6_NO_2_)_2_SbF, 17. (4‐HPy)(Sb_2_OF_4_), 18. Na_2_(4‐HPyH)_2_(PTS)·H_2_O, 19. [HPyClB(OH)_2_](NO_3_)) (d) Section of optical indicatrix for the (001) plane of **I** at 550 nm. The arrows refer to the maximum and minimum in‐plane refractive indices. (e) Theoretically calculated refractive indices of **I**. (f) Triaxial ellipsoid of three principal refractive indices of **I**. (g) Single crystals of **I** were immersed in water at room temperature for 7 days. (h) The interference color of the tested single crystal **I** at different temperatures.

To determine the birefringence value of **I**, we conducted the polarized microscope measurements using a NIKON ECLIPSE LV100N POL polarized microscope. A schematic of the experimental setup is illustrated in Figure 3a. Upon adjusting the Berek compensator, the tested **I** crystal exhibited complete extinction, yielding an optical path difference of *R* = 1.33 µm (Figure 3b) with a measured sample thickness of 4.3 µm (Figure S11). Single‐crystal XRD analysis confirmed that the examined surface corresponds to the (001) crystallographic plane (Figure 3b), which is also the sole plane used for measurement. Based on the birefringence calculation formula [48], the experimental in‐plane birefringence of **I** is about Δ*n*(001)_exp_ = 0.31@550 nm. This result is close to the first‐principles calculated value Δ*n*(001)_cal_ = 0.37@550 nm (Figure 3d), thereby validating the reliability of our computational approach. Considering that **I** crystallize in the orthorhombic system, compound **I** belongs to an optically biaxial crystal. The three optical principal axes *X*, *Y*, and *Z* are correlated to the crystallographic axes *a*, *b*, and *c* through the matrix of $\left(\begin{array}{c} X\\ Y\\ Z \end{array}\right)=\left(\begin{array}{ccc} 1 & 0 & 0\\ 0 & 1 & 0\\ 0 & 0 & 1 \end{array}\right)\;\left(\begin{array}{c} a\\ b\\ c \end{array}\right)$, that is, *n_a_ = n_x_*, *n_b_ = n_y_*, and *n_c_ = n_z_*. As shown in Figure 3e, the refractive indices follow the relationship *n_z_* < *n_x_* < *n_y_*, consistent with our structural analyses, indicating the strongest optical anisotropy along the crystallographic *b* axis. Accordingly, the overall birefringence of **I**, defined as the maximum difference among principal refractive indices, should be Δ*n* = *n_y_* – *n_z_ =* 0.52@550 nm (Figure 3e,f). Combined with its high thermal stability, **I** stands out as a rare example that simultaneously achieves giant birefringence and high thermal stability (Figure 3c; Table S7), positioning it in the top‐right quadrant of Figure 3c (birefringence above 0.5 & thermal stability above 900 K).

To investigate the birefringent performance of compound **I** under various environmental conditions, we first examined its stability in water. As shown in Figure 3g, the interference color of the measured crystal remains unchanged after one week of immersion, demonstrating high stability in water environments. We then examined the thermal stability of birefringence across a wide temperature range (123–373 K). As evidenced in Figure 3h, the interference color is highly stable throughout all the tested temperatures. In addition, the thermal expansion coefficients of **I** were measured by using temperature‐dependent single‐crystal XRD from 100 to 500 K. It can be seen that **I** possesses relatively low thermal expansion, with the volume thermal expansion coefficient of *γ* = 43.86 × 10^−6^ K^−1^ and axial thermal expansion coefficients of *α_a_* = 16.49 × 10^−6^ K^−1^, *α_b_* = 7.39 × 10^−6^ K^−1^, and *α_c_* = 19.64 × 10^−6^ K^−1^ (Figure S12). These results indicate that compound **I** maintains robust birefringent performance across a broad temperature range. This enhanced water and thermal stability may be attributed to the overall crystal structure of **I**.

**FIGURE 4:**
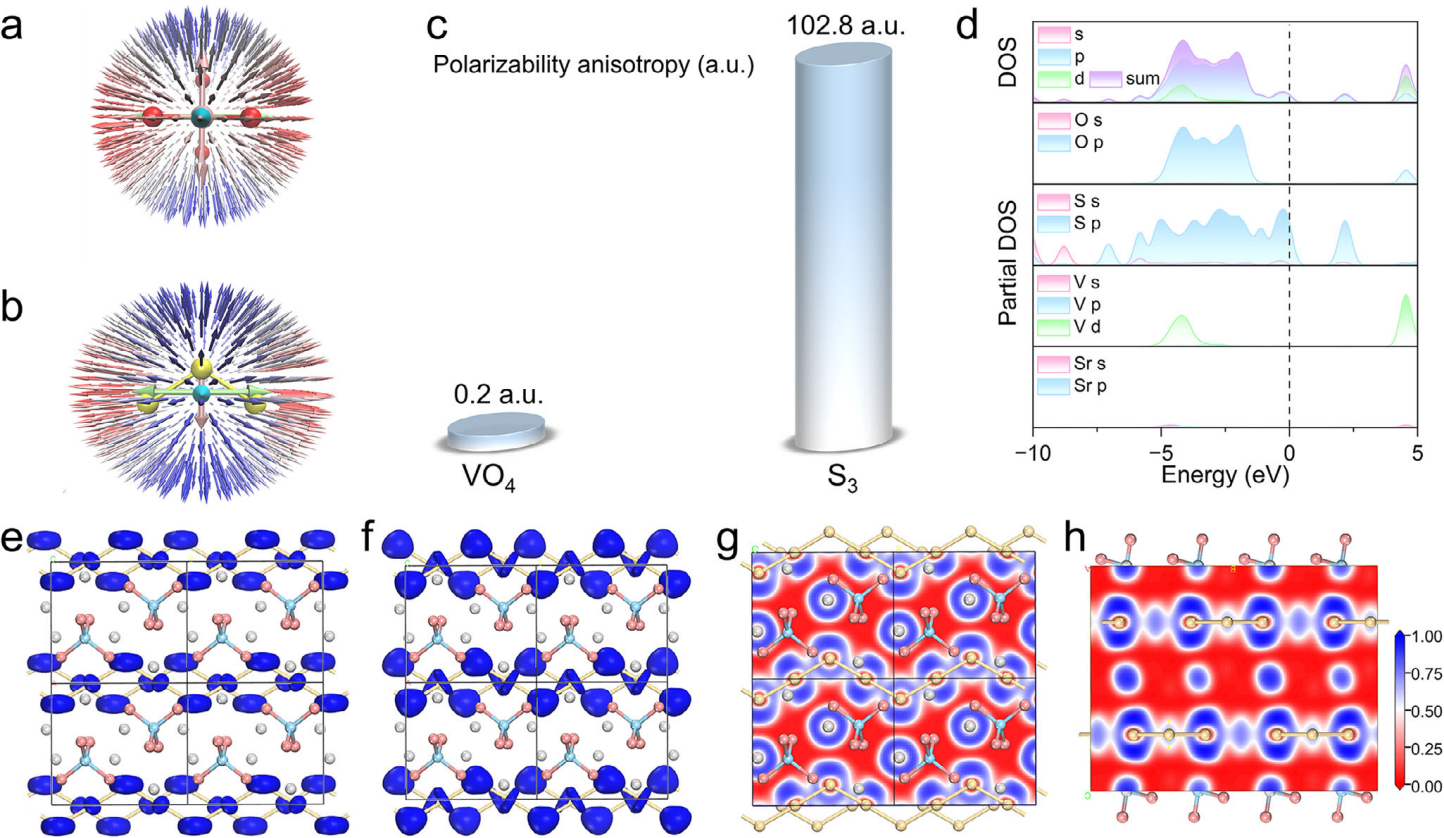
Theoretical calculations of **I**. (a, b) Unit sphere representations of polarizability of (VO_4_)^3−^ and (S_3_)^2−^. (c) Polarizability anisotropy of (VO_4_)^3−^ and (S_3_)^2−^. (d) Total and partial DOS of **I**. (e, f) The HOMO and LUMO of **I**. (g, h) The 2D ELF slices along and perpendicular to the (S_3_)^2−^ chains. Iso‐value increases from red to blue, with the maximum scaled to 1.00. Grey, cyan, gold, and pink spheres represent Sr, V, S, and O atoms, respectively.

To deeply investigate structure‐property relationships, a flexible dipole model was first adopted to calculate the flexibility index (*F*) in (VO_4_)^3−^ tetrahedra and linear (S_3_)^2−^ groups [49]. The S─S bonds in the linear (S_3_)^2−^ groups exhibit an evidently larger flexibility index of *F* = 4.33 than the V─O bonds (*F* = 0.28) in (VO_4_)^3−^ tetrahedra, indicating that linear (S_3_)^2−^ groups possess greater degree of flexibility than the (VO_4_)^3−^ tetrahedra, in accordance with our initial expectations. It also means that S─S bonds are much more susceptible to polarization under external perturbation. We further evaluated the polarizability tensors of rigid (VO_4_)^3−^ and flexible (S_3_)^2−^ [50, 51]. As summarized in Table S8, the polarizability tensor of rigid (VO_4_)^3−^ is nearly isotropic with *α_x__x_* = 85.6 a.u., *α_yy_* = 85.5 a.u., and *α_zz_* = 85.5 a.u. In contrast, the polarizability tensor of flexible (S_3_)^2−^ exhibits pronounced anisotropy, with a significantly larger component along the chain direction (*α_x__x_* = 237.9 a.u.) compared to other directions (*α_yy_* = 127.5 a.u. and *α_zz_* = 145.1 a.u.). The unit sphere representations of polarizability of rigid (VO_4_)^3−^ and flexible (S_3_)^2−^ were visualized to demonstrate this difference [50, 51, 52, 53]. As shown in Figure 4a,b, the nearly spherical distribution of rigid (VO_4_)^3−^ confirms its isotropic polarizability nature, whereas the elongated shape along the (S_3_)^2−^ chain indicates significant polarizability anisotropy. Quantitatively, the polarizability anisotropy of (S_3_)^2−^ (102.8 a.u.) far exceeds that of (VO_4_)^3−^ (0.2 a.u.) (Figure 4c), underscoring the critical role of flexible (S_3_)^2−^ in enhancing linear optical birefringence.

Systematic first‐principles calculations of compound **I** were subsequently performed using the CASTEP package [54, 55]. As demonstrated in Figure S13, **I** exhibits a direct band gap of 1.87 eV, which aligns reasonably with the experiment value 2.30 eV due to the well‐known underestimation by generalized gradient approximation functionals [56]. Figure 4d presents the density of states (DOS) and partial DOS of **I**. It can be clearly seen that the valence band maximum and conduction band minimum near the Fermi level originate primarily from S 2p states. These findings further reinforce that electronic transitions of flexible (S_3_)^2−^ in determining the optical properties of compound **I**. The highest occupied molecular orbital (HOMO) and lowest unoccupied molecular orbital (LUMO) were calculated to better visualize the contributions of flexible (S_3_)^2−^ in the crystal structure of **I**. As shown in Figure 4e,f, both HOMO and LUMO are composed of S 2p orbitals. From the aforementioned analyses, it is evident that flexible (S_3_)^2−^ are responsible for the excellent birefringent performance of compound **I**. Moreover, the electron localization function (ELF) projected onto crystal planes parallel and perpendicular to the quasi‐1D (S_3_)^2−^ chains reveals direction‐dependent variations in electron cloud density around these units. The parallel arrangement of these quasi‐1D (S_3_)^2−^ chains in the crystal structure facilitates the effective superposition of their anisotropic polarizabilities, ultimately leading to large optical birefringence.

## 3 Conclusion

In summary, by implementing a rigid‐flexible coupling strategy, we have developed a novel non‐*π*‐conjugated birefringent crystal, Sr_4_(VO_4_)_2_S_3_, in which highly anisotropic, flexible (S_3_)^2−^ units are combined with rigid (VO_4_)^3−^ tetrahedra. Notably, Sr_4_(VO_4_)_2_S_3_ exhibits a giant birefringence of 0.52@550 nm, surpassing all commercial birefringent crystals, as well as numerous reported birefringent crystals consisting of *π*‐conjugated units. Furthermore, Sr_4_(VO_4_)_2_S_3_ maintains robust birefringence under exposure to air and water and across a broad temperature range, underscoring its practical stability. First‐principles calculations reveal that (S_3_)^2−^ groups are significantly more flexible than the (VO_4_)^3−^ tetrahedra, contributing to the microscopic origin of large polarizability anisotropy. The flexible (S_3_)^2−^ groups align parallel to each other within the crystal structure, further enhancing the material's polarizability anisotropy, while the rigid (VO_4_)^3−^ tetrahedra reinforce structural robustness. It paves the way for next‐generation linear optical materials by exploring other rigid frameworks (e.g., (BO_4_)^5−^, (SiO_4_)^4−^, (PO_4_)^3−^, and (SO_4_)^2−^) coupled with diverse flexible anisotropic units (e.g., (BO_2_)^−^, (S_2_)^2−^, (Se_2_)^2−^, and (Se_3_)^2−^).

## Conflicts of Interest

The authors declare no conflicts of interest.

## Supporting information




**Supporting File 1**: advs74948‐sup‐0001‐SuppMat.docx.

**Supporting File 2**: advs74948‐sup‐0002‐Data.zip.

## Data Availability

The data that support the findings of this study are available in the Supporting Information of this article.
