# Supplementary material for: Rigid‐Flexible Coupling Units Enable Robust Large Birefringence
Source: Adv Sci (Weinh). 2026 Mar 26;13(32):e74948. doi: 10.1002/advs.74948 (PMC13252608; doi:10.1002/advs.74948)
Supplement: Supplementary file 1 — Supporting File 1: advs74948‐sup‐0001‐SuppMat.docx. [file ADVS-13-e74948-s001.docx]

Supplementary Information

**Rigid-Flexible Coupling Units Enable Robust Large Birefringence**

Jie Zhou,^1,2,4^ Yanqiang Li,^1,3,^* Ji Qi,^1^ Weiqi Huang,^1^ Liangmeng Zhu,^1^ Zhiyong Bai,^1^ Yang Zhou,^3^ Junhua Luo,^1,4,^* and Sangen Zhao^3,^*

^1^State Key Laboratory of Functional Crystals and Devices, Fujian Institute of Research on the Structure of Matter, Chinese Academy of Sciences, Fuzhou, Fujian 350002, China

^2^College of Chemistry, Fuzhou University, Fuzhou, Fujian 350108, China

^3^Quantum Science Center of Guangdong-Hong Kong-Macao Greater Bay Area, Shenzhen, Guangdong 518045, China

^4^Fujian College, University of Chinese Academy of Sciences, Fuzhou, Fujian 350002, China

*Correspondence to: liyanqiang@quantumsc.cn; jhluo@fjirsm.ac.cn; zhaosangen@quantumsc.cn

CONTENTS

[Reagents 4](#_Toc223546547)

[Synthesis 4](#_Toc223546548)

[Single-Crystal Structure Determination 5](#_Toc223546549)

[Powder XRD Analysis 5](#_Toc223546550)

[Thermal Stability 5](#_Toc223546551)

[Semiquantitative microprobe analysis 6](#_Toc223546552)

[XPS Analysis 6](#_Toc223546553)

[FTIR Analysis 6](#_Toc223546554)

[Raman Analysis 6](#_Toc223546555)

[Polarized Raman Analysis 6](#_Toc223546556)

[UV–Vis–NIR Diffuse Reflectance Spectroscopy 7](#_Toc223546557)

[Birefringence Tests 7](#_Toc223546558)

[Theoretical Calculations 8](#_Toc223546559)

[Flexibility Index 9](#_Toc223546560)

[Figure S1. Polyhedron (a) Sr2O_6_S_3_ and (b) Sr1O_6_S_4_. 10](#_Toc223546561)

[Figure S2. Quasi-1D [S_3_]_∞_ chains. 10](#_Toc223546562)

[Figure S3. Experimental and simulated powder XRD patterns of Sr_4_(VO_4_)_2_S_3_. 11](#_Toc223546563)

[Figure S4. Single crystals of Sr_4_(VO_4_)_2_S_3_ were immersed in water at room temperature for 7 days. 11](#_Toc223546564)

[Figure S5. TG and DTA curves of Sr_4_(VO_4_)_2_S_3_. 12](#_Toc223546565)

[Figure S6. Powder XRD patterns of Sr_4_(VO_4_)_2_S_3_ samples after heating to 940 K and 980 K under flowing N_2_, along with its calculated powder XRD pattern. 12](#_Toc223546566)

[Figure S7. EDS result of Sr_4_(VO_4_)_2_S_3_. 13](#_Toc223546567)

[Figure S8. Raman spectrum of Sr_4_(VO_4_)_2_S_3_. 13](#_Toc223546568)

[Figure S9. FTIR of Sr_4_(VO_4_)_2_S_3_. 14](#_Toc223546569)

[Figure S10. The UV-Vis-NIR diffuse reflectance spectrum of Sr_4_(VO_4_)_2_S_3_. The inset represents the experiment bandgap. 14](#_Toc223546570)

[Figure S11. The thickness of Sr_4_(VO_4_)_2_S_3_ crystal used for birefringence measurements. 15](#_Toc223546571)

[Figure S12. Temperature variation of the lattice parameters (*V*, *a*, *b*, and *c*-axes) of Sr_4_(VO_4_)_2_S_3_. 15](#_Toc223546572)

[Figure S13. Electronic band structure of Sr_4_(VO_4_)_2_S_3_. 16](#_Toc223546573)

[Table S1. Crystal data and structure refinement for Sr_4_(VO_4_)_2_S_3_. 17](#_Toc223546574)

[Table S2. Atomic coordinates (× 10^4^) and equivalent isotropic displacement parameters (Å^2^ × 10^3^) for Sr_4_(VO_4_)_2_S_3_. 18](#_Toc223546575)

[Table S3. Anisotropic displacement parameters (Å^2^ × 10^3^) for Sr_4_(VO_4_)_2_S_3_. 19](#_Toc223546576)

[Table S4. Selected bond lengths (Å) for Sr_4_(VO_4_)_2_S_3_. 20](#_Toc223546577)

[Table S5. Selected bond angles (°) for Sr_4_(VO_4_)_2_S_3_. 21](#_Toc223546578)

[Table S6. Atomic Occupancy for Sr_4_(VO_4_)_2_S_3_. 25](#_Toc223546579)

[Table S7. Comparison of the birefringence and stable temperature of Sr_4_(VO_4_)_2_S_3_ with those of all commercial birefringent crystals, recently reported famous birefringent crystals and optical crystals composed of tetrahedral structural units. 26](#_Toc223546580)

[Table S8. Static polarizability (*α*) of (VO_4_)^3-^ and (S_3_)^2-^ (a.u.). 31](#_Toc223546581)

[References 32](#_Toc223546582)

**Reagents**

All the reagents including SrO (Rhawn, AR), V_2_O_5_ (Rhawn, 99.0%), V (Leyan, 99.0%, 325Mesh) and S (Aladdin, 99.5%) without further purification.

# Synthesis

Polycrystalline powder of Sr_4_(VO_4_)_2_S_3_ was synthesized via high-temperature solid-state reaction under anhydrous and anaerobic conditions. A stoichiometric mixture of SrO (3 mmol), V_2_O_5_ (0.6 mmol), V (0.3 mmol), and S (3 mmol) was thoroughly ground inside a dry glovebox filled with inert nitrogen gas, where the levels of oxygen and water vapor were maintained below 0.1 ppm. The homogenized mixture was then transferred to an alumina crucible. This crucible was placed inside a quartz tube (Ф 11 mm × 15 cm), which was subsequently sealed using sealing film within the glovebox. The sealed tube was then removed from the glovebox. The quartz tube containing the reactants was evacuated to a high vacuum of 10-20 Pa and flame-sealed. The sealed tube was then placed in a programmable muffle furnace. The temperature was raised to 673 K over 6 h, held at this temperature for 16 h, further increased to 973 K over 5 h, and maintained at 973 K for 4 days. Subsequently, the furnace was cooled to 873 K over 22 h. After the reaction, the furnace was turned off and allowed to cool naturally to room temperature.

Single crystals of Sr_4_(VO_4_)_2_S_3_ were grown via high-temperature flux synthesis under anhydrous and anaerobic conditions. A mixture of SrO (3 mmol), V (1 mmol), and S (4 mmol) was thoroughly ground inside a dry glovebox filled with inert nitrogen gas, where the levels of oxygen and water vapor were maintained below 0.1 ppm. The homogenized mixture was transferred to an alumina crucible. This crucible was placed inside a quartz tube (Ф 11 mm × 15 cm), which was subsequently sealed using sealing film within the glovebox. The sealed tube was then removed from the glovebox. The quartz tube containing the reactants was evacuated to a high vacuum of 10-20 Pa and flame-sealed. The sealed tube was placed in a programmable muffle furnace. The temperature was raised to 1023 K over 23 h, held at this temperature for 4 days, and then cooled to 573 K over 30 h. After the reaction, the furnace was turned off and allowed to cool naturally to room temperature. Upon completion of the reaction, numerous orange crystals were found adhered to the walls of the alumina crucible.

# Single-Crystal Structure Determination

Orange block-shaped crystals of Sr_4_(VO_4_)_2_S_3_ were selected for single-crystal X-ray diffraction (XRD) analysis. Diffraction data for Sr_4_(VO_4_)_2_S_3_ were collected at 100 K using graphite-monochromated Mo-K*α* radiation (*λ* = 0.71073 Å) on a Rigaku XtaLAB Synergy-R diffractometer. Intensity data were collected and processed using CrysAlisPro software for unit-cell refinement and data reduction. Using Olex2,^1^ the crystal structure was solved with the SHELXS^2^ structure solution program using Direct Methods and refined with the SHEXL^3^ refinement package using Least Squares minimization. Final refinements include anisotropic displacement parameters. The final crystal structure was verified by the ADDSYM algorithm from the PLATON program,^4^ and no higher symmetry was found. Detailed crystal parameters, data collection, and structure refinement were summarized in Table S1. The atomic coordinates and equivalent isotropic displacement parameters were listed in Table S2. The anisotropic displacement parameters were listed in Table S3. Selected bond lengths and bond angles were presented in Table S4 and S5.

# Powder XRD Analysis

Powder XRD investigations on polycrystalline Sr_4_(VO_4_)_2_S_3_ were carried out at room temperature on a Rigaku MiniFlex 600 diffractometer equipped with Mo-Ka radiation. A scanning step width of 0.02° and scanning rate of 5° min-1 was applied to record the patterns in the 2θ range of 10-80°. The measured powder XRD pattern matches well with the one calculated from single-crystal XRD analysis.

# Thermal Stability

The thermal stability of Sr_4_(VO_4_)_2_S_3_ was investigated by the thermogravimetric (TG) and differential thermal analysis (DTA) on a NETZSCH STA 449F3 simultaneous thermal analyzer instrument. About 7.16 mg Sr_4_(VO_4_)_2_S_3_ powders were placed into an Al_2_O_3_ crucible, heated at a rate of 10 K min^−1^ from room temperature to 1273 K. The measurement was carried out in an atmosphere of N_2_ flowing.

# Semiquantitative microprobe analysis

Semiquantitative microprobe analysis on the Sr_4_(VO_4_)_2_S_3_ crystal was conducted using a field emission scanning electron microscope (SEM) (Hitachi SU8010) equipped with energy dispersive X-ray spectroscopy (EDS). The EDS data was collected on clean surfaces of the samples, confirming the presence of Sr, V, S and O elements. In addition, the SEM elemental mapping of the Sr_4_(VO_4_)_2_S_3_ single crystal was also collected on the field emission SEM.

# XPS Analysis

The X-ray photoelectron spectroscopy (XPS) was operated on the ESCALAB 250Xi XPS instrument by using Al K*α* radiation as the source.

# FTIR Analysis

Fourier transform infrared spectroscopy (FTIR) in the wavenumber range of 4000–400 cm^−1^ was recorded on the Bruker Vertex 70 infrared spectrometer.

# Raman Analysis

Raman spectroscopy between 1500 and 100 cm^−1^ range was collected on a LabRAM HR Evolution Raman microscope (HORIBA Scientific) with a solid-state laser corresponding to the green light (*λ* = 532 nm).

**Polarized Raman Analysis**

Raman measurements were performed using a double spectrometer (LabRAM Odyssey) equipped with a liquid nitrogen cooled CCD detector. A 532 nm laser (Excelsior, Spectra-Physics) was focused onto the sample with a 50× objective and a power of 10%.

A half-wave plate was placed between the dichroic mirrors and the laser, allowing rotation of the polarization directions of the incident beam. The incident laser beam is polarized, and a analyzer was placed just before the spectrometer entrance, allowing the investigation of the scattered light polarization along directions parallel and perpendicular to the incident light polarization. Throughout the paper, these two polarization configurations are called, respectively, the parallel and cross polarization configurations and are represented in Figure 2a, where the angle *θ* between the incident light polarization and the crystalline b direction is also defined. Angle-dependent Raman data were collected for every 15° of half-waveplate rotation. The half-waveplate rotate every 15° from 0°-360°, allowing the measurement of the angular dependence of the Raman intensities in both polarization configurations.

# UV–Vis–NIR Diffuse Reflectance Spectroscopy

The UV–Vis–NIR diffuse reflection data was recorded at room temperature using a powdered BaSO_4_ sample as a standard (100% reflectance) on a PerkinElmer Lamda-950 UV–Vis–NIR spectrophotometer. The scanning wavelength range is from 400 nm to 800 nm. Absorption (*K*/*S*) data was calculated from the following Kubelka–Munk function:^5^

$$\begin{aligned} \text{F}\text{(}\text{R}\text{)=(1-}\text{R}\text{)}\text{2}\text{/(2}\text{R}\text{)=}\text{K}\text{/}\text{S}\#\text{Eq}\left( \text{1} \right) \end{aligned}$$

*R* is the reflectance, *K* is the absorption, and *S* is the scattering.

# Birefringence Tests

The birefringence of Sr_4_(VO_4_)_2_S_3_ was characterized by the polarized method under the polarized microscope (Nikon ECLIPSE LV100N POL) equipped with a Berek compensator. The wavelength of the light source was *λ* = 550 nm. The relative error is small enough because of the clear boundary lines of the first-, second-, and third-order interference color. In order to improve the accuracy of the birefringence, the small and transparent Sr_4_(VO_4_)_2_S_3_ crystals were chosen. The formula for calculating the birefringence is listed below:^6, 7^

$$\begin{aligned} \text{R}\text{=}\text{|}\text{n}_{\text{e}}\text{-}\text{n}_{\text{o}}\text{|}\text{×}\text{T}\text{=∆}\text{n}\text{×}\text{T}\#\text{Eq}\left( \text{2} \right) \end{aligned}$$

Here, *R* represents the optical path difference, Δ*n* is the birefringence, and *T* denotes the thickness of the tested crystal.

# Theoretical Calculations

First-principles calculations were conducted using the CASTEP software,^8^ a plane-wave pseudopotential package^9^ on the basis of the density functional theory (DFT).^10^ The exchange-correlation energy was described by the generalized gradient approximation (GGA) scheme of Perdew–Burke–Ernzerhof (PBE) functional, as implemented in the CASTEP code.^11^ Before the calculation, atomic positions in the unit cell have been fully geometrically optimized with the aid of the Broyden-Fletcher-Goldfarb-Shannon (BFGS) algorithm.^12^ Norm-conserving pseudopotentials were employed to simulate the ion-electron interactions for each atomic specie with following valence configurations: Sr 5*s*^2^; V 3*d*^3^4*s*^2^; S 3*s*^2^3*p*^4^; O 2*s*^2^2*p*^4^.^13^ A cutoff energy of 750 eV and the Monkhorst–Pack^14^ *k*-point meshes (4 × 1 × 6) spacing less than 0.03 Å^−1^ in the Brillouin zone were chosen for the calculation.

*Ab initio* calculations were conducted by the Gaussian 16 program.^15^ Final theoretical analyses were carried out with Multiwfn 3.8 (dev) code,^16^ and the isosurface graphs were rendered by the VMD program.^17^

The anisotropy of the polarizability tensor was defined by the static polarizability tensor, according to the following Eq (3).^18^ Detailed static polarizability and polarizability anisotropy for different groups were shown in Table S8. Herein, we choose the most commonly used one, a.u., as the unit of the polarizability anisotropy.

$$\begin{aligned} \text{Δ}\text{α}\text{=}\sqrt{\text{[(}\text{α}_{\text{xx}}\text{-}\text{α}_{\text{yy}}\text{)}^{\text{2}}\text{+(}\text{α}_{\text{xx}}\text{-}\text{α}_{\text{zz}}\text{)}^{\text{2}}\text{+(}\text{α}_{\text{yy}}\text{-}\text{α}_{\text{zz}}\text{)}^{\text{2}}\text{+6(}\text{α}_{\text{xy}}^{\text{2}}\text{+}\text{α}_{\text{xz}}^{\text{2}}\text{+}\text{α}_{\text{yz}}^{\text{2}}\text{)}\text{]/2}}\#\text{Eq}\left( \text{3} \right) \end{aligned}$$

where *α* represents the static polarizability tensor, *δ* represents anisotropy of the polarizability tensor.

# Flexibility Index

The formula to calculate the flexibility index is expressed as follows:^19^

$$\begin{aligned} \text{F}\text{=}\frac{\text{exp}\left[ \left( \text{R}_{\text{0}}\text{-}\text{R} \right)\text{/}\text{B} \right]}{\left( \sqrt{\text{C}_{\text{a}}}\text{+}\sqrt{\text{C}_{\text{b}}} \right)^{\text{2}}\text{/}\text{R}^{\text{2}}}\#\text{Eq}\left( \text{4} \right) \end{aligned}$$

where the numerator represents the bond valence charge derived from the bond valence model,^20^ in which *R* is the length of the concerned bond, *R*_0_ is the ideal bond length when the bonding atoms perfectly contribute a unit valence,^21^ and *B* is an empirical constant (typically 0.37 Å).^22^ *C_a_* and *C_b_* are the charges of the atomic cores formed by the bonding atoms losing the outer-shell electrons.


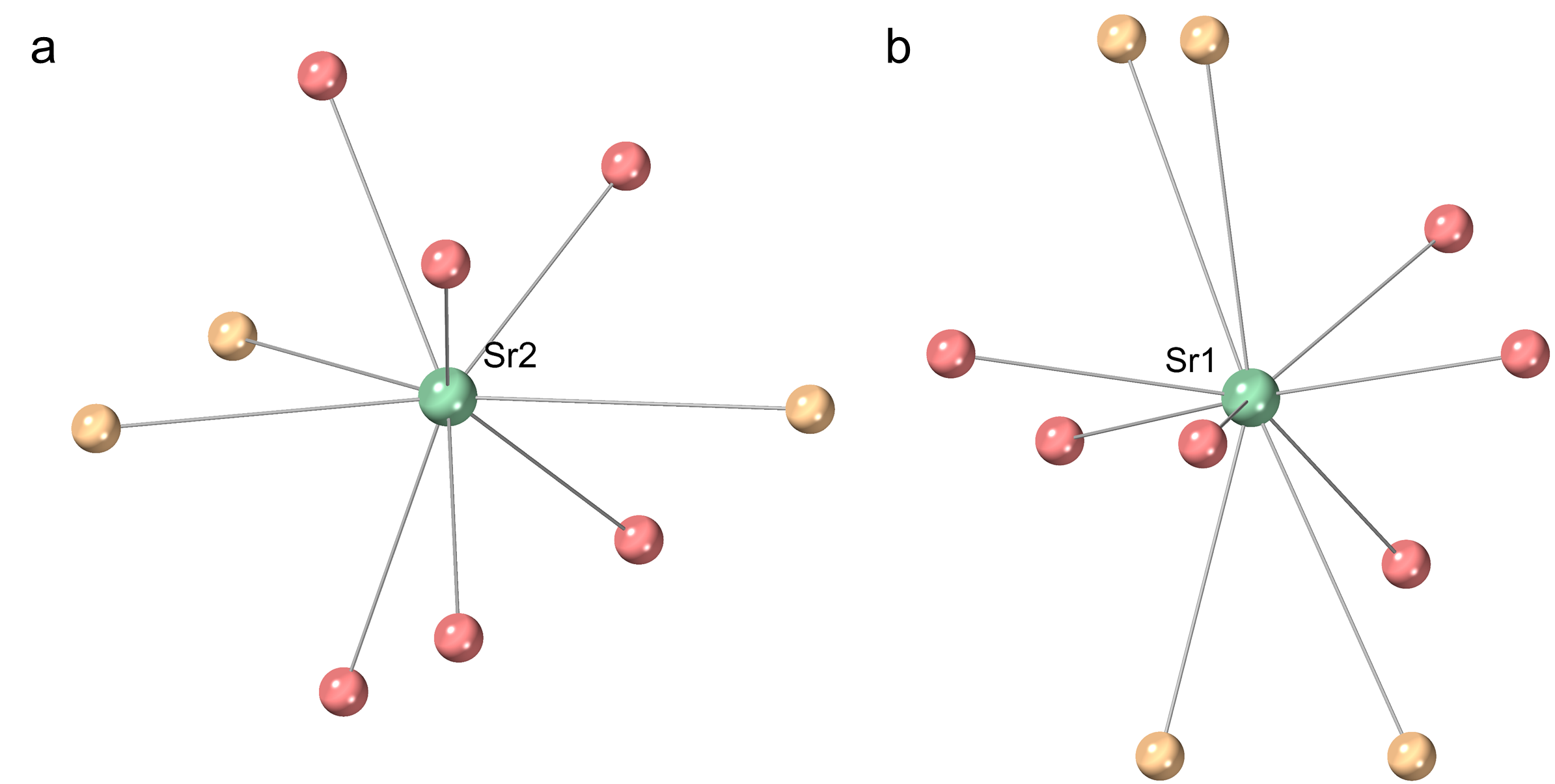


# Figure S1. Polyhedron (a) Sr2O_6_S_3_ and (b) Sr1O_6_S_4_.


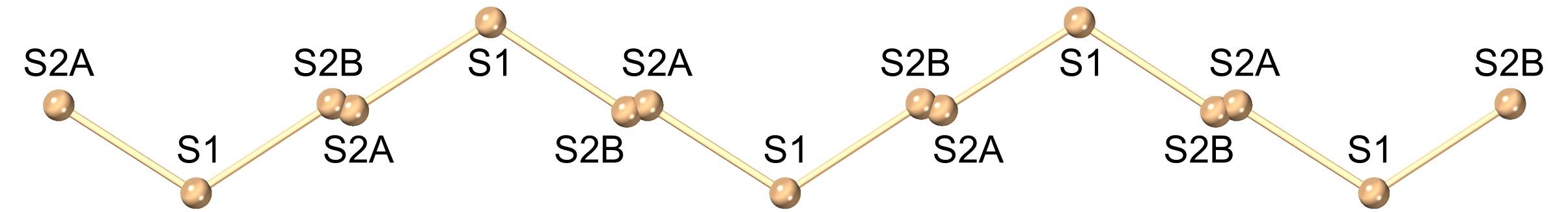


# Figure S2. Quasi-1D [S_3_]_∞_ chains.

The S1 atom lies on a symmetry-restricted special position and therefore shows symmetry-induced half occupancy (occ. = 0.50), while the S2 atom is split into two alternative sites (S2A/S2B; occ. = 0.50/0.50). Application of the relevant symmetry operations generates the crystallographically equivalent atoms and forms the (S_3_)^2−^ units. Owing to the *b*-glide symmetry, these partially occupied (S_3_)^2−^ units appear in the average structure as alternating orientations along the *b*-axis.


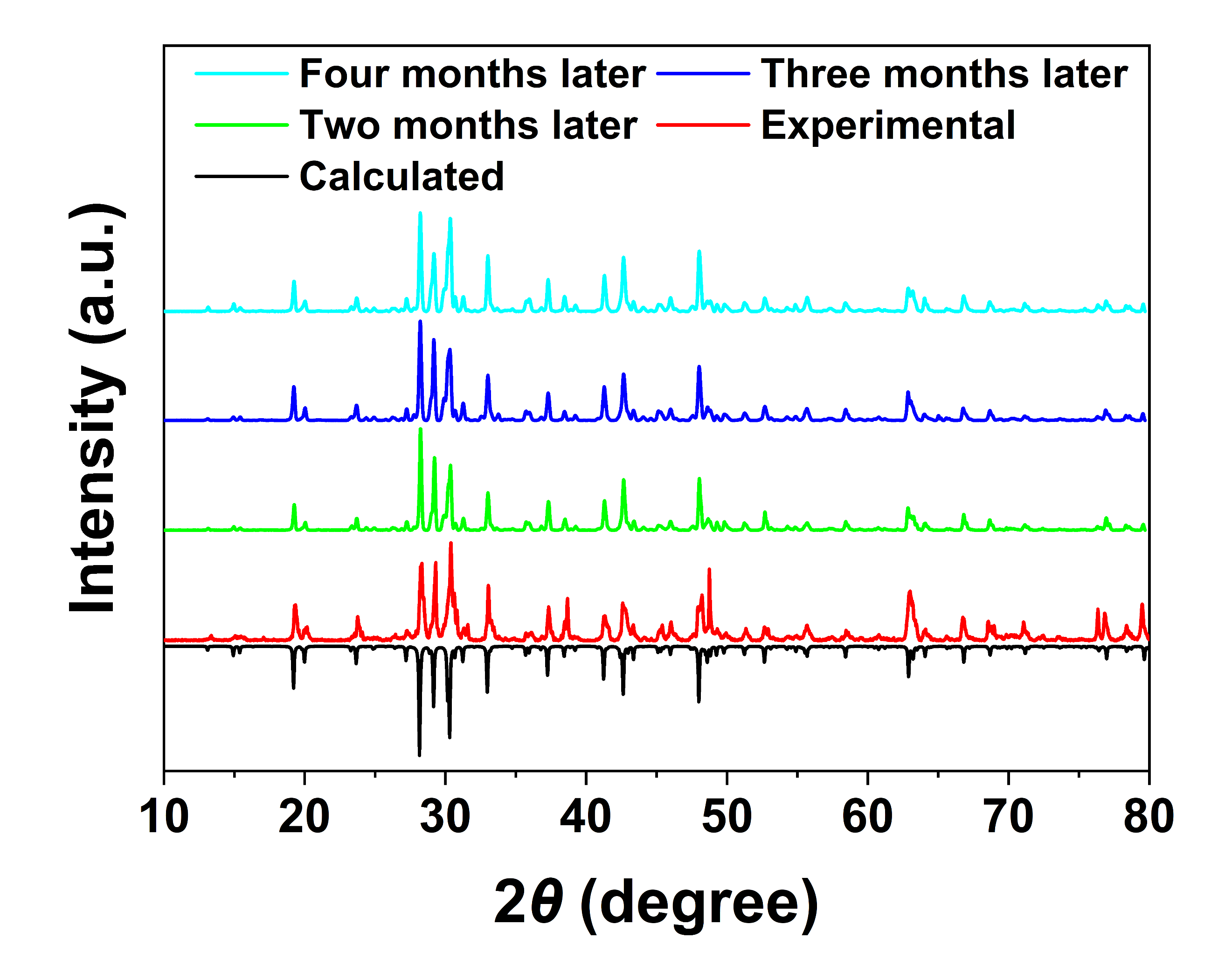


# Figure S3. Experimental and simulated powder XRD patterns of Sr_4_(VO_4_)_2_S_3_.


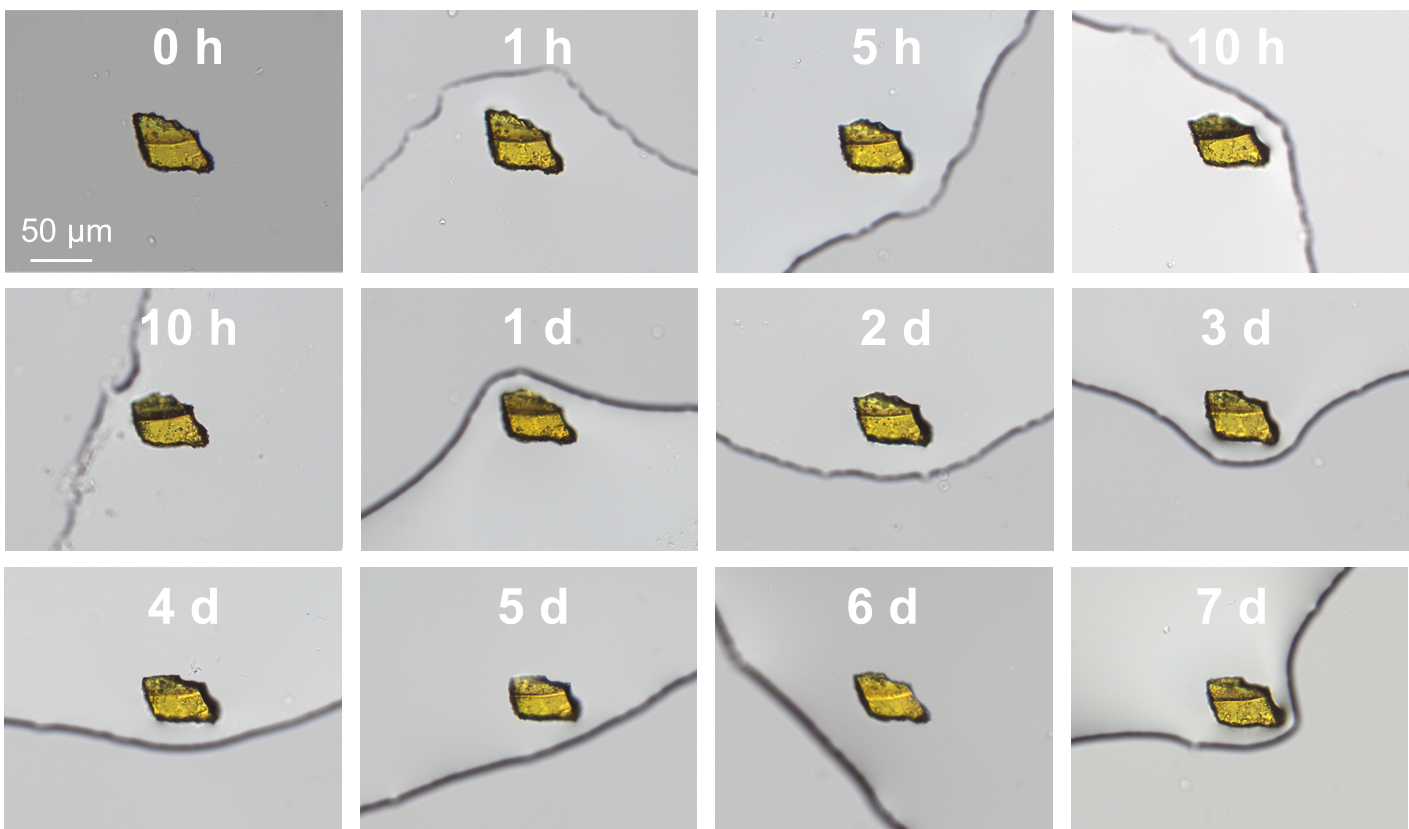


**Figure S4.** Single crystals of Sr_4_(VO_4_)_2_S_3_ were immersed in water at room temperature for 7 days.


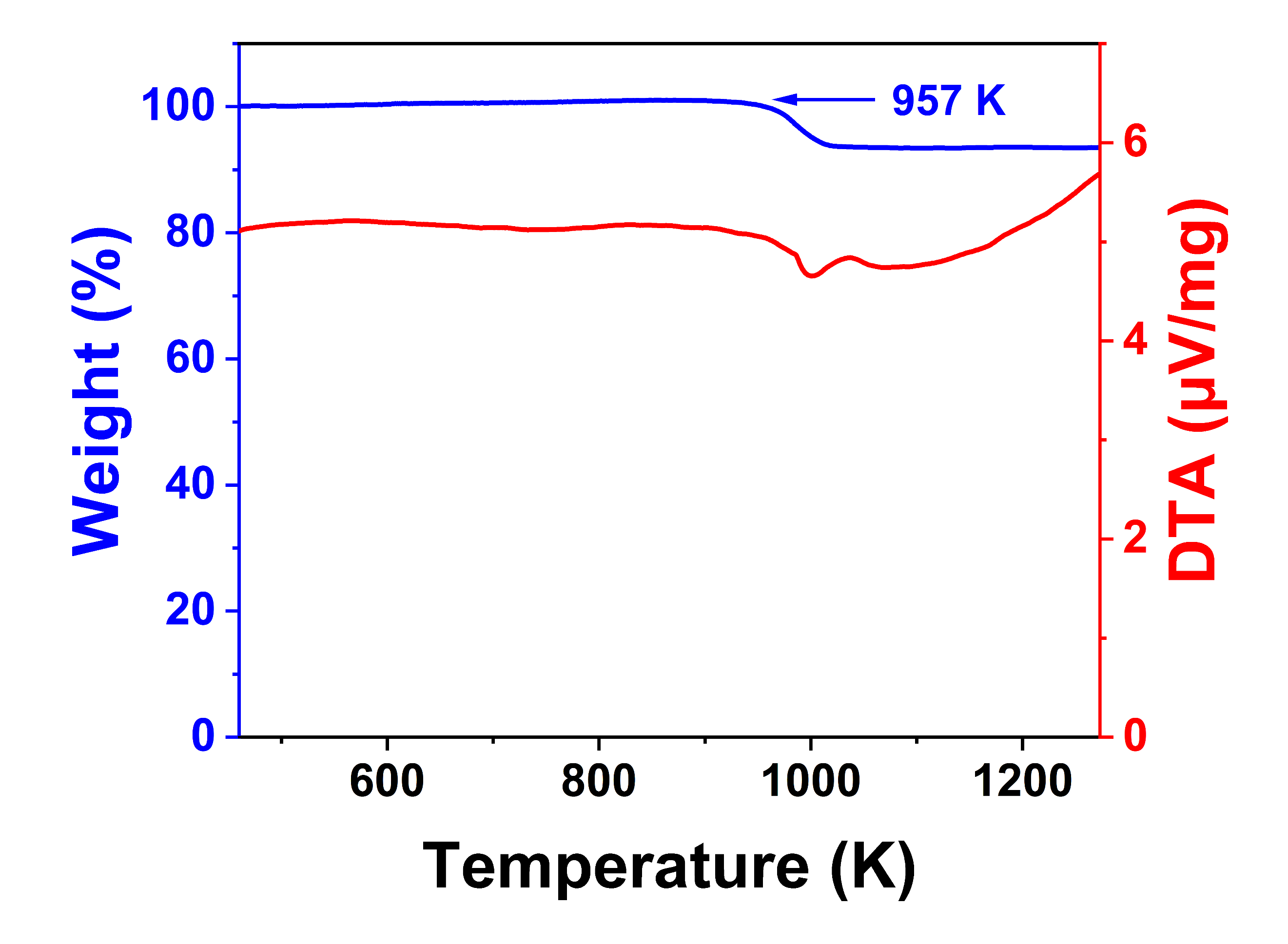


# Figure S5. TG and DTA curves of Sr_4_(VO_4_)_2_S_3_.


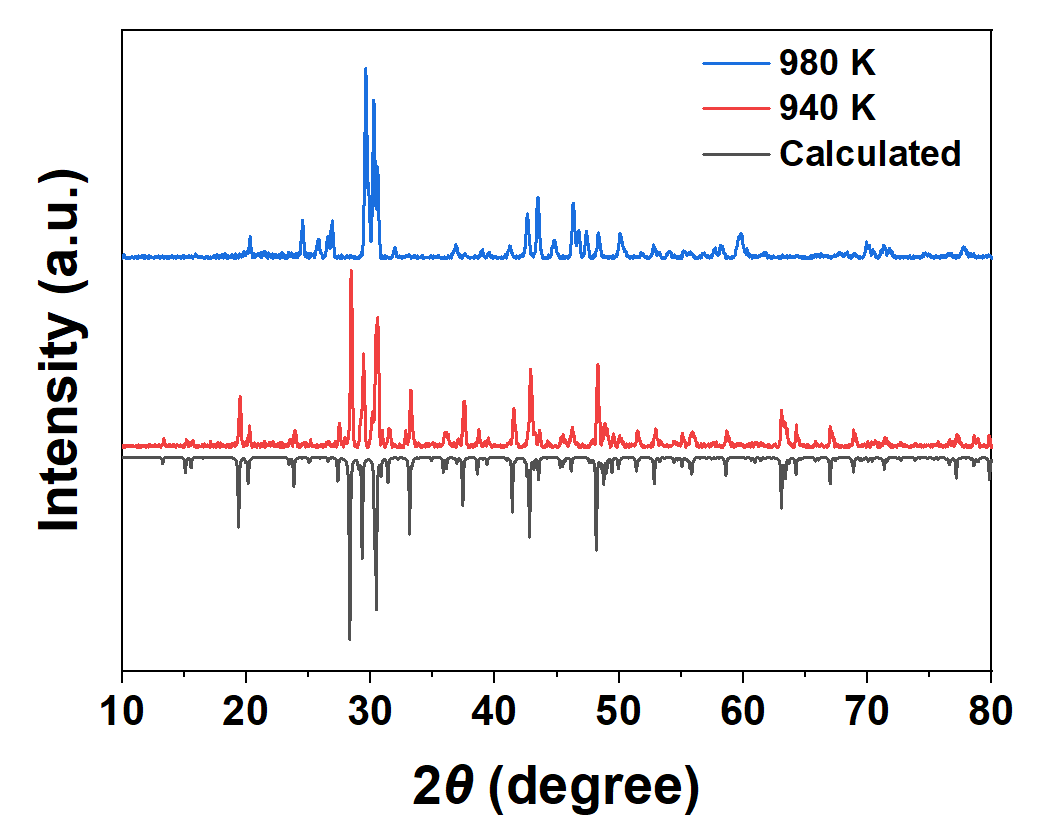


# Figure S6. Powder XRD patterns of Sr_4_(VO_4_)_2_S_3_ samples after heating to 940 K and 980 K under flowing N_2_, along with its calculated powder XRD pattern.


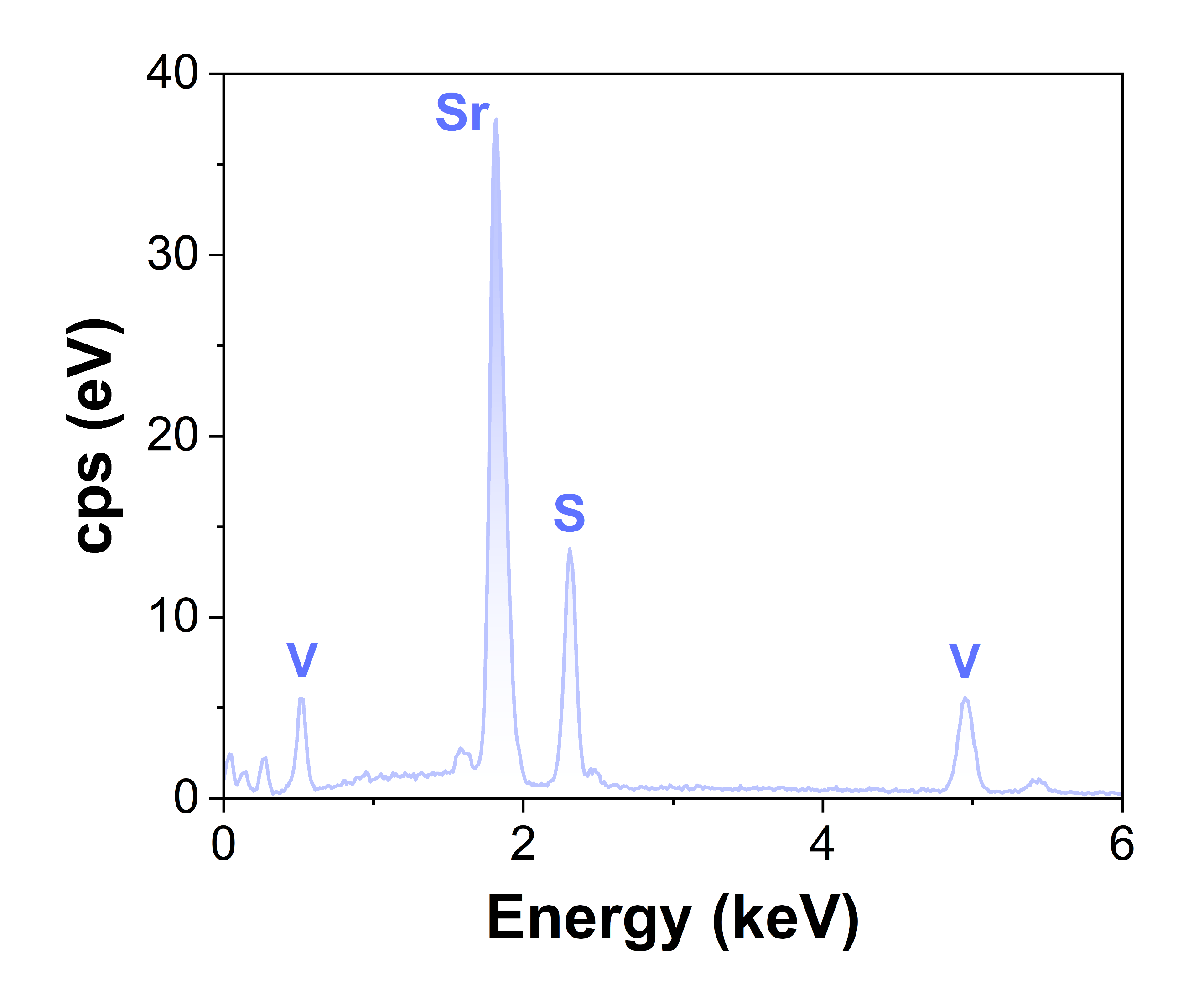


# Figure S7. EDS result of Sr_4_(VO_4_)_2_S_3_.


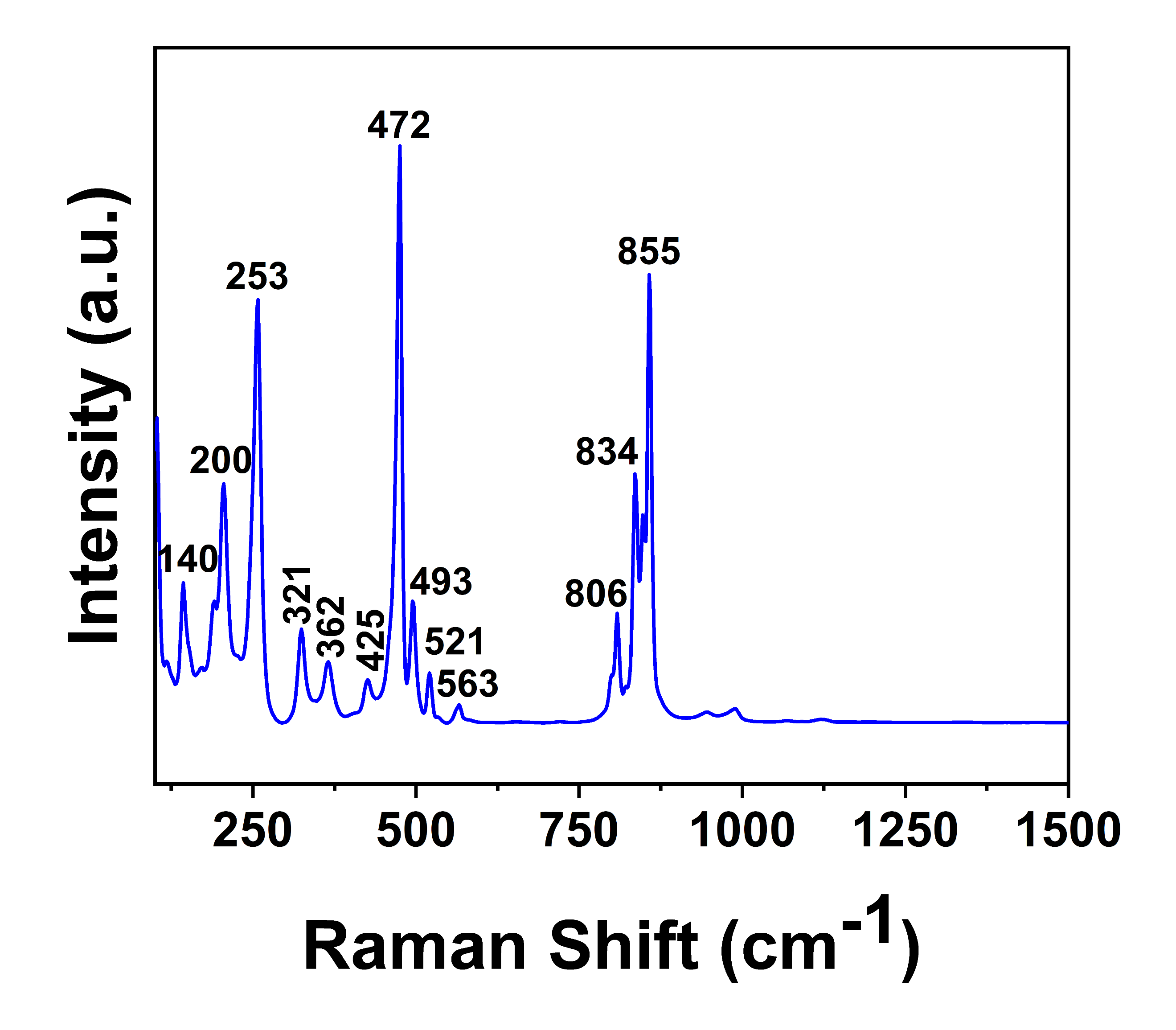


# Figure S8. Raman spectrum of Sr_4_(VO_4_)_2_S_3_.


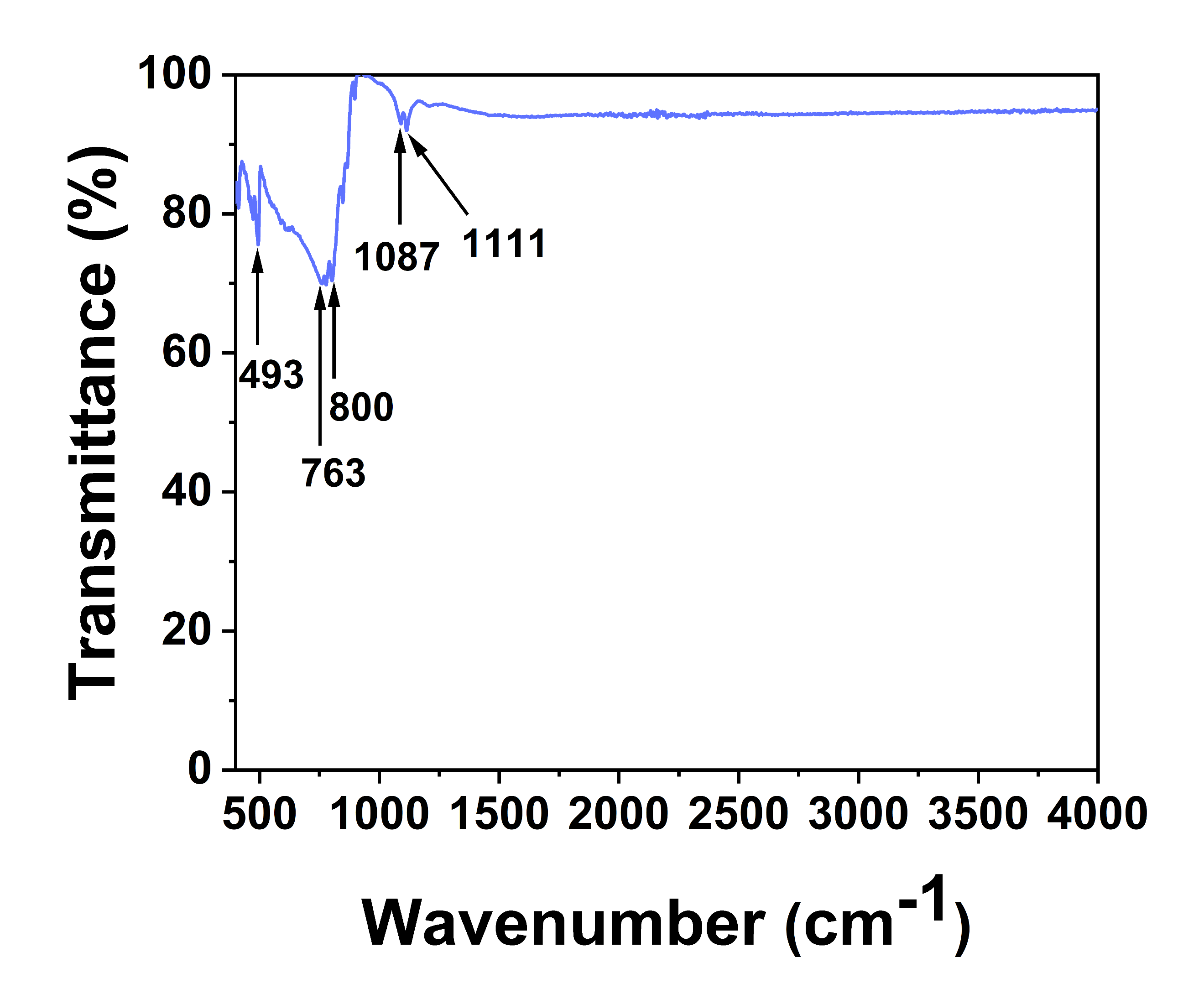


# Figure S9. FTIR of Sr_4_(VO_4_)_2_S_3_.


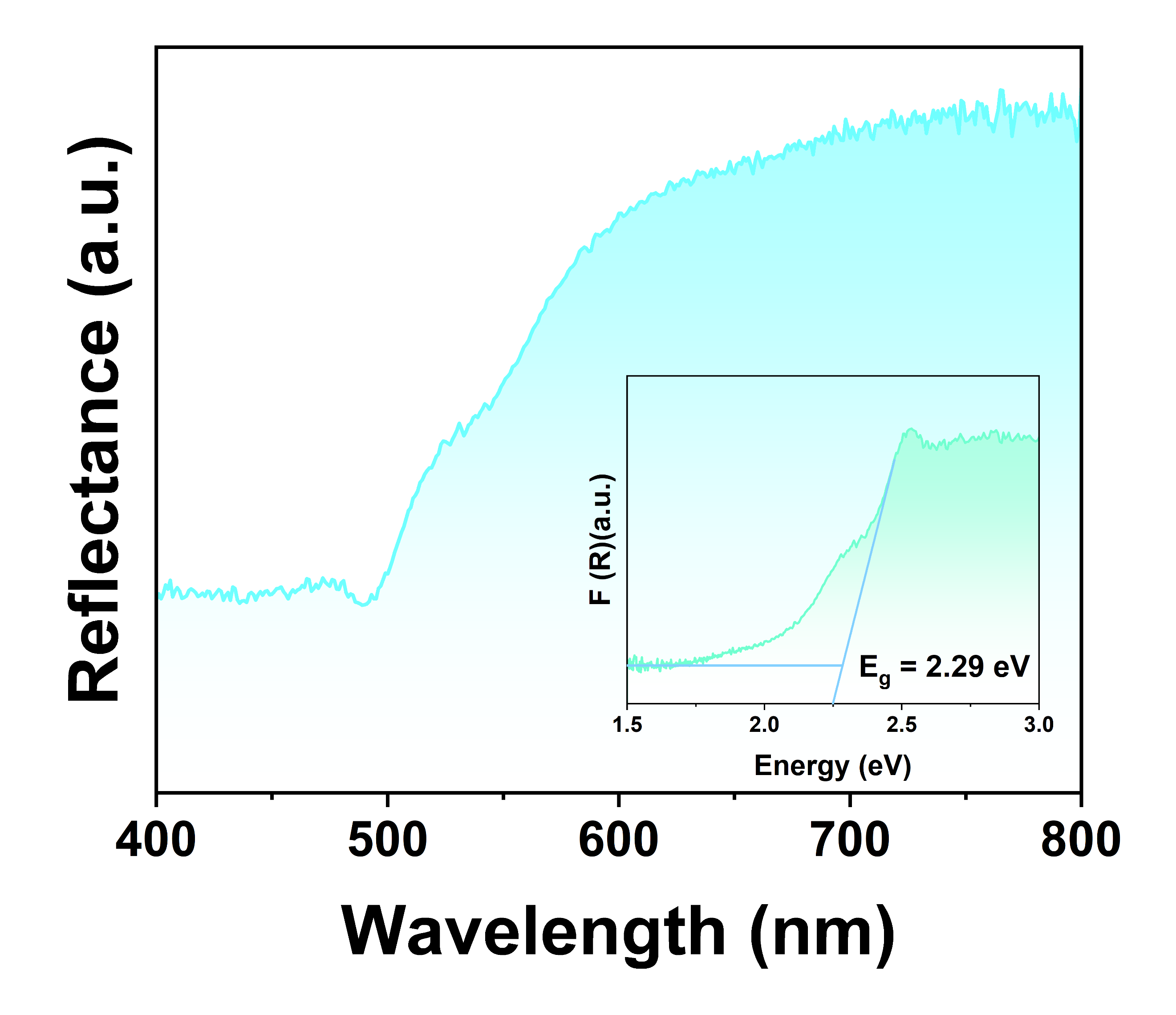


# Figure S10. The UV-Vis-NIR diffuse reflectance spectrum of Sr_4_(VO_4_)_2_S_3_. The inset represents the experiment bandgap.


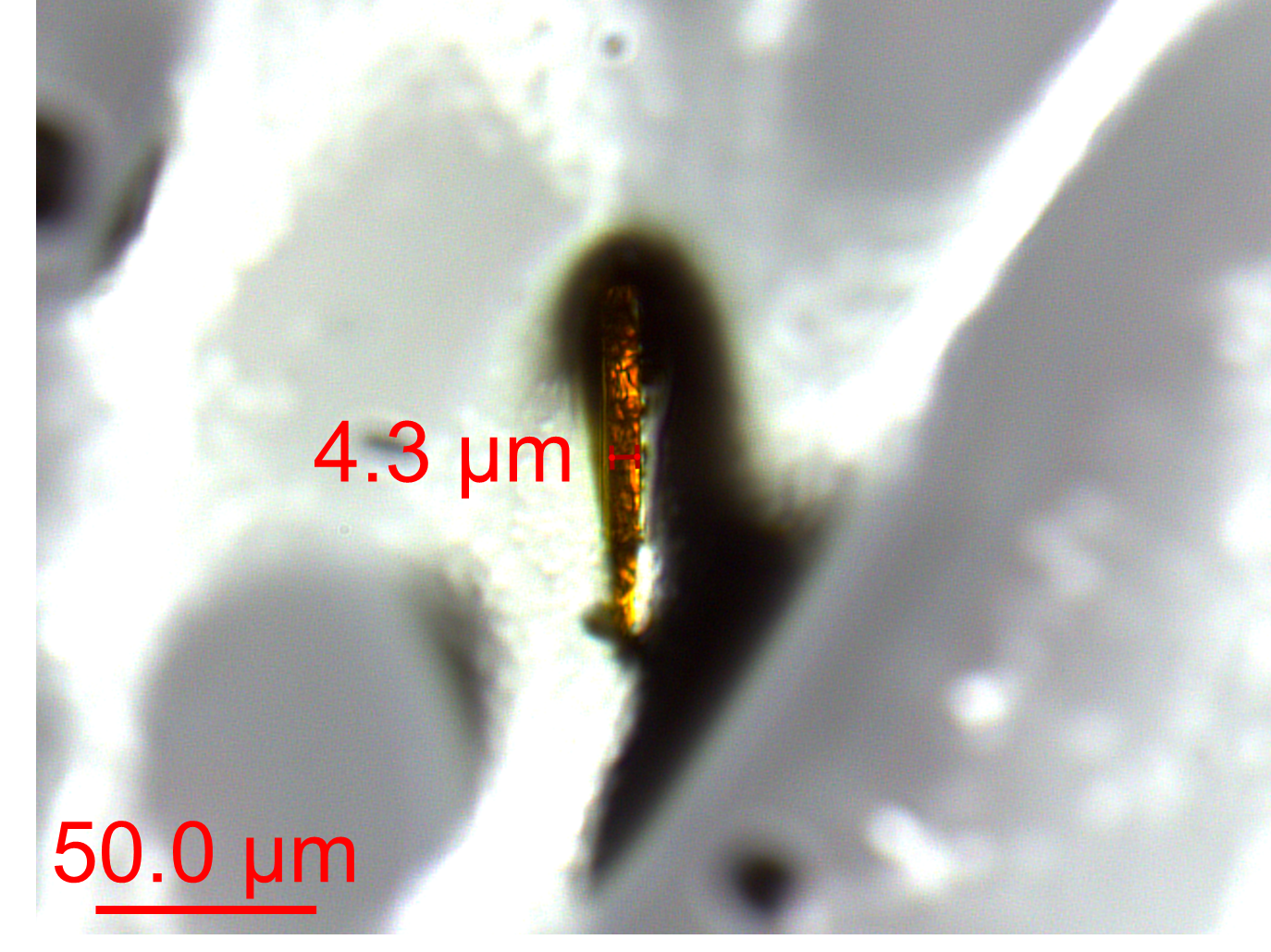


**Figure S11.** The thickness of Sr_4_(VO_4_)_2_S_3_ crystal used for birefringence measurements.


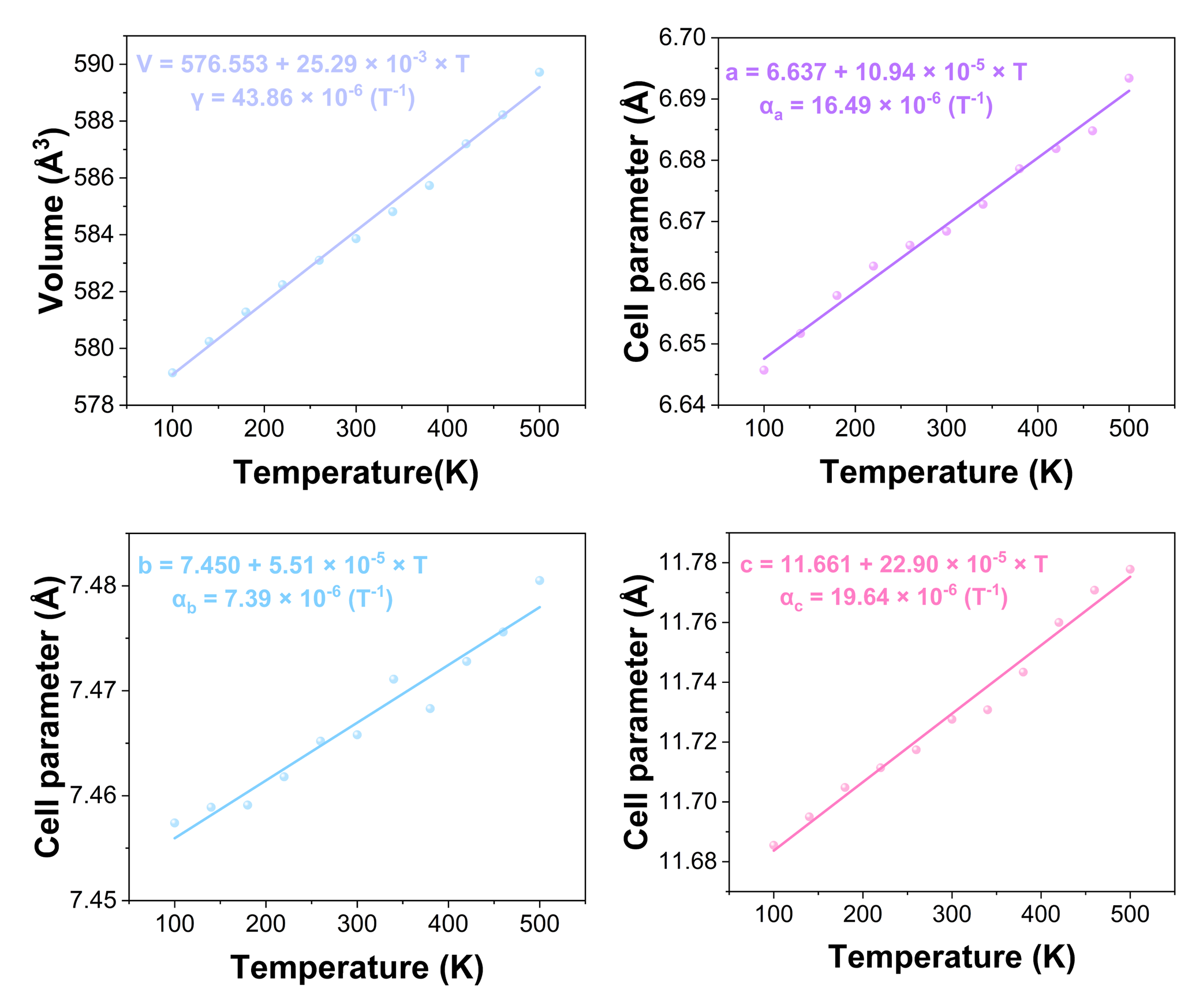


**Figure S12.** Temperature variation of the lattice parameters (*V*, *a*, *b*, and *c*-axes) of Sr_4_(VO_4_)_2_S_3_.


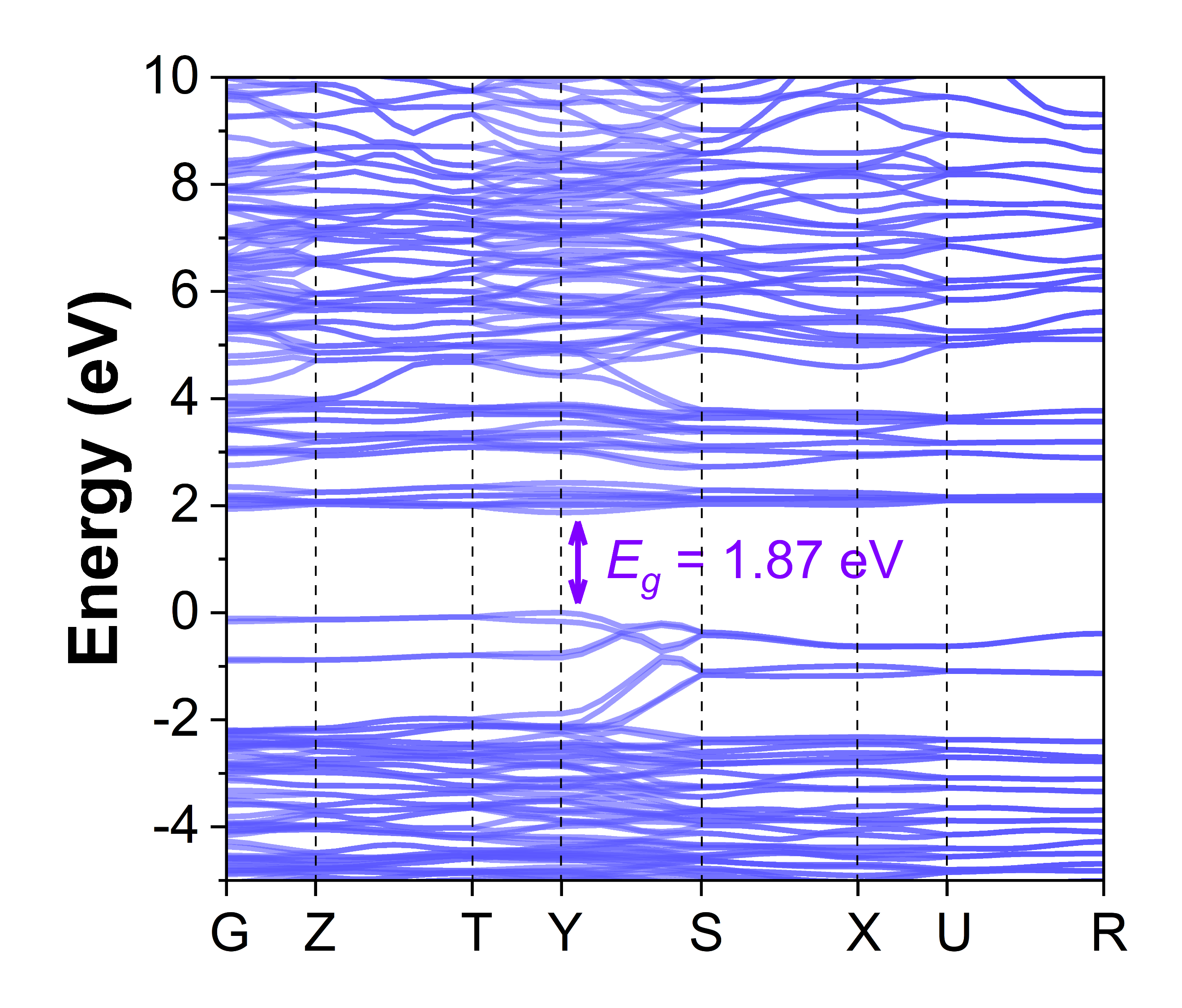


**Figure S13.** Electronic band structure of Sr_4_(VO_4_)_2_S_3_.

**Table S1.** Crystal data and structure refinement for Sr_4_(VO_4_)_2_S_3_.

| Empirical formula | Sr_4_(VO_4_)_2_S_3_ |
| --- | --- |
| Formula weight | 676.54 |
| Temperature/K | 100.00(10) |
| Crystal system | orthorhombic |
| Space group | *Pbcm* |
| *a*/Å | 6.6556(2) |
| *b*/Å | 7.4628(2) |
| *c*/Å | 11.6900(3) |
| *α*/° | 90 |
| *β*/° | 90 |
| *γ*/° | 90 |
| Volume/Å^3^ | 580.64(3) |
| Z | 2 |
| *ρ*(g cm^–3^) | 3.870 |
| *μ*/mm^–1^ | 20.342 |
| *F*(000) | 620.0 |
| Radiation | Mo K*α* (*λ* = 0.71073) |
| 2*θ* range for data collection/° | 6.122 to 81.864 |
| Index ranges | -11 ≤ *h* ≤ 12, -13 ≤ *k* ≤ 12, -21 ≤ *l* ≤ 19 |
| Reflections collected | 6727 |
| Independent reflections | 1945 [*R_int_* = 0.0229, *R_sigma_* = 0.0247] |
| Data/restraints/parameters | 1945/28/54 |
| Goodness-of-fit on F^2^ | 1.060 |
| Final *R* indexes [I≥2*σ*(I)]^[a]^ | *R*_1_ = 0.0261*, wR*_2_ = 0.0670 |
| Final *R* indexes [all data]^[a]^ | *R*_1_ = 0.0341, *wR*_2_ = 0.0695 |
| Largest diff. peak/hole / e Å^–3^ | 1.91/-1.52 |

^[a]^*wR*_1_ = Σ||F_o_|–F_c_||/Σ|F_o_| and *wR*_2_ = [Σ*w*(F_o_^2^–F_c_^2^)^2^/Σ*w*F_o_^4^]^1/2^ for F_o_^2^ > 2*σ*(F_o_^2^).

# Table S2. Atomic coordinates (× 10^4^) and equivalent isotropic displacement parameters (Å^2^ × 10^3^) for Sr_4_(VO_4_)_2_S_3_.

| Atom | *x* | *y* | *z* | *U*_eq_^[a]^ |
| --- | --- | --- | --- | --- |
| Sr1 | 1306.0(3) | 2500 | 5000 | 6.07(5) |
| Sr2 | 6242.4(3) | 4639.0(3) | 7500 | 8.64(5) |
| V1 | 3667.8(5) | 7500 | 5000 | 5.25(7) |
| S1 | 8378(2) | 9522.1(13) | 7500 | 16.1(2) |
| O1 | 2132.2(18) | 5744.9(15) | 4689.3(11) | 10.13(19) |
| O2 | 5199.1(17) | 7202.8(17) | 6171.8(11) | 9.74(19) |
| S2A | 10048(5) | 7190(3) | 7500 | 10.6(4) |
| S2B | 9925(5) | 6836(3) | 7500 | 8.7(3) |

^[a]^*U*_eq_ is defined as one-third of the trace of the orthogonalized *U*_ij_ tensor.

# Table S3. Anisotropic displacement parameters (Å^2^ × 10^3^) for Sr_4_(VO_4_)_2_S_3_.

| Atom | *U*_11_ | *U*_22_ | *U*_33_ | *U*_23_ | *U*_13_ | *U*_12_ |
| --- | --- | --- | --- | --- | --- | --- |
| Sr1 | 5.07(8) | 6.25(9) | 6.89(9) | 1.06(5) | 0 | 0 |
| Sr2 | 13.08(9) | 7.86(9) | 4.99(8) | 0 | 0 | -3.05(6) |
| V1 | 5.06(13) | 5.97(14) | 4.72(14) | -0.23(9) | 0 | 0 |
| S1 | 9.3(4) | 12.8(5) | 26.1(7) | 0 | 0 | -1.3(4) |
| O1 | 8.5(4) | 8.2(4) | 13.7(5) | -1.4(4) | 2.0(4) | -1.3(3) |
| O2 | 8.6(4) | 13.3(5) | 7.2(4) | 0.6(4) | -0.2(3) | 2.6(4) |
| S2A | 10.9(6) | 11.8(8) | 9.2(5) | 0 | 0 | -0.3(6) |
| S2B | 9.4(6) | 9.9(7) | 6.8(5) | 0 | 0 | 0.3(6) |

# Table S4. Selected bond lengths (Å) for Sr_4_(VO_4_)_2_S_3_.

| Atom1 | Atom2 | Length(Å) |  | Atom1 | Atom2 | Length(Å) |
| --- | --- | --- | --- | --- | --- | --- |
| Sr1 | S1^1^ | 3.2958(4) |  | Sr2 | O2^8^ | 2.5600(12) |
| Sr1 | S1^2^ | 3.2958(5) |  | Sr2 | O2 | 2.5600(12) |
| Sr1 | O1^3^ | 2.5096(12) |  | Sr2 | O2^6^ | 2.5761(13) |
| Sr1 | O1 | 2.5096(12) |  | Sr2 | O2^2^ | 2.5761(12) |
| Sr1 | O1^4^ | 2.6616(12) |  | Sr2 | S2A | 3.168(3) |
| Sr1 | O1^5^ | 2.6616(12) |  | Sr2 | S2A^9^ | 3.072(3) |
| Sr1 | O2^6^ | 2.7086(12) |  | Sr2 | S2B^9^ | 3.299(3) |
| Sr1 | O2^1^ | 2.7086(12) |  | Sr2 | S2B | 2.949(3) |
| Sr1 | S2A^2^ | 3.0670(11) |  | V1 | O1 | 1.7007(12) |
| Sr1 | S2A^1^ | 3.0670(11) |  | V1 | O1^10^ | 1.7007(12) |
| Sr1 | S2B^1^ | 3.0753(10) |  | V1 | O2 | 1.7218(12) |
| Sr1 | S2B^2^ | 3.0753(10) |  | V1 | O2^10^ | 1.7218(12) |
| Sr2 | S1^2^ | 3.0763(14) |  | S1 | S2A | 2.065(3) |
| Sr2 | O1^1^ | 2.7932(13) |  | S1 | S2B^11^ | 2.064(3) |
| Sr2 | O1^7^ | 2.7932(13) |  |  |  |  |

^1^1-X,1-Y,1-Z; ^2^1-X,-1/2+Y,3/2-Z; ^3^+X,1/2-Y,1-Z; ^4^-X,1-Y,1-Z; ^5^-X,-1/2+Y,+Z; ^6^1-X,-1/2+Y,+Z; ^7^1-X,1-Y,1/2+Z; ^8^+X,+Y,3/2-Z; ^9^2-X,-1/2+Y,3/2-Z; ^10^+X,3/2-Y,1-Z; ^11^2-X,1/2+Y,3/2-Z

# Table S5. Selected bond angles (°) for Sr_4_(VO_4_)_2_S_3_.

| Atom1 | Atom2 | Atom3 | Angle(*˚*) |  | Atom1 | Atom2 | Atom3 | Angle(*˚*) |
| --- | --- | --- | --- | --- | --- | --- | --- | --- |
| S1^1^ | Sr1 | S1^2^ | 172.68(5) |  | O2^6^ | Sr2 | S2A^7^ | 83.08(5) |
| O1^3^ | Sr1 | S1^1^ | 107.43(3) |  | O2^9^ | Sr2 | S2A^7^ | 131.51(4) |
| O1^4^ | Sr1 | S1^1^ | 73.05(4) |  | O2^1^ | Sr2 | S2A | 136.20(4) |
| O1^4^ | Sr1 | S1^2^ | 113.66(4) |  | O2^1^ | Sr2 | S2A^7^ | 83.08(5) |
| O1 | Sr1 | S1^1^ | 70.89(3) |  | O2^6^ | Sr2 | S2A | 136.20(4) |
| O1^5^ | Sr1 | S1^2^ | 73.05(4) |  | O2 | Sr2 | S2A | 76.57(5) |
| O1^5^ | Sr1 | S1^1^ | 113.66(4) |  | O2^9^ | Sr2 | S2A | 76.57(5) |
| O1^3^ | Sr1 | S1^2^ | 70.89(3) |  | O2 | Sr2 | S2A^7^ | 131.51(4) |
| O1 | Sr1 | S1^2^ | 107.43(3) |  | O2 | Sr2 | S2B^7^ | 133.13(4) |
| O1 | Sr1 | O1^3^ | 154.69(6) |  | O2^6^ | Sr2 | S2B^7^ | 80.82(5) |
| O1^3^ | Sr1 | O1^4^ | 130.05(4) |  | O2^6^ | Sr2 | S2B | 134.59(4) |
| O1^5^ | Sr1 | O1^4^ | 61.42(5) |  | O2^1^ | Sr2 | S2B | 134.59(4) |
| O1 | Sr1 | O1^4^ | 74.53(4) |  | O2^9^ | Sr2 | S2B | 79.04(5) |
| O1 | Sr1 | O1^5^ | 130.05(4) |  | O2^1^ | Sr2 | S2B^7^ | 80.82(5) |
| O1^3^ | Sr1 | O1^5^ | 74.53(4) |  | O2 | Sr2 | S2B | 79.04(5) |
| O1^3^ | Sr1 | O2^2^ | 87.94(4) |  | O2^9^ | Sr2 | S2B^7^ | 133.13(4) |
| O1^4^ | Sr1 | O2^6^ | 135.20(4) |  | S2A^7^ | Sr2 | S1^1^ | 141.87(6) |
| O1^5^ | Sr1 | O2^2^ | 135.20(4) |  | S2A^7^ | Sr2 | S2A | 73.440(5) |
| O1^5^ | Sr1 | O2^6^ | 140.09(4) |  | S2A^7^ | Sr2 | S2B^7^ | 2.84(10) |
| O1 | Sr1 | O2^6^ | 87.94(4) |  | S2A | Sr2 | S2B^7^ | 76.28(10) |
| O1 | Sr1 | O2^2^ | 70.10(4) |  | S2B | Sr2 | S2B^7^ | 73.134(8) |
| O1^3^ | Sr1 | O2^6^ | 70.10(4) |  | O1 | V1 | O1^11^ | 106.12(8) |
| O1^4^ | Sr1 | O2^2^ | 140.09(4) |  | O1 | V1 | O2^11^ | 106.56(6) |
| O1 | Sr1 | S2A^1^ | 105.97(5) |  | O1 | V1 | O2 | 115.24(6) |
| O1^5^ | Sr1 | S2A^2^ | 69.79(6) |  | O1^11^ | V1 | O2 | 106.56(6) |
| O1^3^ | Sr1 | S2A^2^ | 105.97(5) |  | O1^11^ | V1 | O2^11^ | 115.25(6) |
| O1^4^ | Sr1 | S2A^1^ | 69.79(6) |  | O2 | V1 | O2^11^ | 107.41(8) |
| O1^3^ | Sr1 | S2A^1^ | 81.58(5) |  | Sr1^2^ | S1 | Sr1^8^ | 124.93(3) |
| O1^5^ | Sr1 | S2A^1^ | 80.81(7) |  | Sr1^8^ | S1 | Sr2^12^ | 86.98(3) |
| Atom1 | Atom2 | Atom3 | Angle(*˚*) |  | Atom1 | Atom2 | Atom3 | Angle(*˚*) |
| O1^4^ | Sr1 | S2A^2^ | 80.81(7) |  | Sr1^2^ | S1 | Sr2^12^ | 86.98(3) |
| O1 | Sr1 | S2A^2^ | 81.58(5) |  | Sr2^10^ | S1 | Sr1^2^ | 94.40(3) |
| O1^3^ | Sr1 | S2B^2^ | 110.57(5) |  | Sr2^10^ | S1 | Sr1^8^ | 94.40(3) |
| O1 | Sr1 | S2B^2^ | 76.43(5) |  | Sr2^10^ | S1 | Sr2^12^ | 176.98(3) |
| O1^4^ | Sr1 | S2B^2^ | 79.71(7) |  | S1^9^ | S1 | Sr1^2^ | 0(10) |
| O1^5^ | Sr1 | S2B^1^ | 79.71(7) |  | S1^9^ | S1 | Sr1^8^ | 0(10) |
| O1^5^ | Sr1 | S2B^2^ | 73.78(6) |  | S1^9^ | S1 | Sr2^12^ | 0(5) |
| O1 | Sr1 | S2B^1^ | 110.57(5) |  | S1^9^ | S1 | Sr2^10^ | 0(10) |
| O1^3^ | Sr1 | S2B^1^ | 76.43(5) |  | S1^9^ | S1 | S2A^12^ | 0(10) |
| O1^4^ | Sr1 | S2B^1^ | 73.78(6) |  | S1^9^ | S1 | S2A | 0(10) |
| O2^2^ | Sr1 | S1^1^ | 110.87(4) |  | S1^9^ | S1 | S2B^12^ | 0(10) |
| O2^6^ | Sr1 | S1^1^ | 62.24(4) |  | S2A | S1 | Sr1^2^ | 65.15(2) |
| O2^2^ | Sr1 | S1^2^ | 62.24(3) |  | S2A | S1 | Sr1^8^ | 65.15(2) |
| O2^6^ | Sr1 | S1^2^ | 110.87(4) |  | S2A^12^ | S1 | Sr1^8^ | 112.05(3) |
| O2^6^ | Sr1 | O2^2^ | 61.64(5) |  | S2A^12^ | S1 | Sr1^2^ | 112.05(3) |
| O2^2^ | Sr1 | S2A^2^ | 76.36(7) |  | S2A^12^ | S1 | Sr2^12^ | 60.84(9) |
| O2^6^ | Sr1 | S2A^1^ | 76.36(7) |  | S2A | S1 | Sr2^10^ | 124.18(10) |
| O2^2^ | Sr1 | S2A^1^ | 137.76(7) |  | S2A^12^ | S1 | Sr2^10^ | 116.14(9) |
| O2^6^ | Sr1 | S2A^2^ | 137.76(7) |  | S2A | S1 | Sr2^12^ | 58.84(9) |
| O2^2^ | Sr1 | S2B^1^ | 136.27(7) |  | S2A | S1 | S2A^12^ | 119.68(6) |
| O2^6^ | Sr1 | S2B^2^ | 136.27(7) |  | Sr1 | O1 | Sr1^4^ | 105.47(4) |
| O2^2^ | Sr1 | S2B^2^ | 74.63(7) |  | Sr1 | O1 | Sr2^2^ | 96.81(4) |
| O2^6^ | Sr1 | S2B^1^ | 74.63(7) |  | Sr1^4^ | O1 | Sr2^2^ | 120.56(5) |
| S2A^2^ | Sr1 | S1^1^ | 146.01(5) |  | V1 | O1 | Sr1^4^ | 96.23(5) |
| S2A^2^ | Sr1 | S1^2^ | 37.66(5) |  | V1 | O1 | Sr1 | 147.56(7) |
| S2A^1^ | Sr1 | S1^2^ | 146.01(5) |  | V1 | O1 | Sr2^2^ | 92.40(5) |
| S2A^1^ | Sr1 | S1^1^ | 37.66(5) |  | Sr2 | O2 | Sr1^2^ | 97.76(4) |
| S2A^2^ | Sr1 | S2A^1^ | 145.83(13) |  | Sr2^10^ | O2 | Sr1^2^ | 124.53(5) |
| S2A^2^ | Sr1 | S2B^2^ | 5.16(7) |  | Sr2 | O2 | Sr2^10^ | 105.25(4) |
| S2A^1^ | Sr1 | S2B^1^ | 5.16(7) |  | V1 | O2 | Sr1^2^ | 95.47(5) |
| Atom1 | Atom2 | Atom3 | Angle(*˚*) |  | Atom1 | Atom2 | Atom3 | Angle(*˚*) |
| S2A^1^ | Sr1 | S2B^2^ | 147.09(3) |  | V1 | O2 | Sr2^10^ | 99.68(5) |
| S2A^2^ | Sr1 | S2B^1^ | 147.09(3) |  | V1 | O2 | Sr2 | 137.68(7) |
| S1^1^ | Sr2 | S2A | 144.69(6) |  | Sr1^2^ | S2A | Sr1^8^ | 144.69(13) |
| S1^1^ | Sr2 | S2B^7^ | 139.02(6) |  | Sr1^8^ | S2A | Sr2^12^ | 101.02(5) |
| O1^8^ | Sr2 | S1^1^ | 112.60(3) |  | Sr1^8^ | S2A | Sr2 | 79.08(6) |
| O1^2^ | Sr2 | S1^1^ | 112.60(3) |  | Sr1^2^ | S2A | Sr2 | 79.08(6) |
| O1^8^ | Sr2 | O1^2^ | 132.76(5) |  | Sr1^2^ | S2A | Sr2^12^ | 101.02(5) |
| O1^8^ | Sr2 | S2A | 75.64(3) |  | Sr2^12^ | S2A | Sr2 | 179.58(11) |
| O1^8^ | Sr2 | S2A^7^ | 68.14(3) |  | S1^9^ | S2A | Sr1^8^ | 77.19(6) |
| O1^2^ | Sr2 | S2A^7^ | 68.14(3) |  | S1^9^ | S2A | Sr1^2^ | 77.19(6) |
| O1^2^ | Sr2 | S2A | 75.64(3) |  | S1 | S2A | Sr1^8^ | 77.19(6) |
| O1^2^ | Sr2 | S2B | 74.64(3) |  | S1 | S2A | Sr1^2^ | 77.19(6) |
| O1^8^ | Sr2 | S2B^7^ | 68.62(3) |  | S1^9^ | S2A | Sr2^12^ | 86.05(8) |
| O1^8^ | Sr2 | S2B | 74.64(3) |  | S1 | S2A | Sr2 | 94.37(13) |
| O1^2^ | Sr2 | S2B^7^ | 68.62(3) |  | S1^9^ | S2A | Sr2 | 94.37(13) |
| O2^1^ | Sr2 | S1^1^ | 66.90(3) |  | S1 | S2A | Sr2^12^ | 86.05(8) |
| O2^9^ | Sr2 | S1^1^ | 75.53(3) |  | S1^9^ | S2A | S1 | 0.00(7) |
| O2 | Sr2 | S1^1^ | 75.53(3) |  | Sr1^2^ | S2B | Sr1^8^ | 143.73(12) |
| O2^6^ | Sr2 | S1^1^ | 66.90(3) |  | Sr1^2^ | S2B | Sr2^12^ | 95.96(5) |
| O2 | Sr2 | O1^2^ | 68.04(4) |  | Sr1^8^ | S2B | Sr2^12^ | 95.96(5) |
| O2^1^ | Sr2 | O1^2^ | 128.61(4) |  | Sr2 | S2B | Sr1^2^ | 82.43(6) |
| O2^9^ | Sr2 | O1^8^ | 68.04(4) |  | Sr2 | S2B | Sr1^8^ | 82.43(6) |
| O2^9^ | Sr2 | O1^2^ | 137.51(4) |  | Sr2 | S2B | Sr2^12^ | 174.43(11) |
| O2^6^ | Sr2 | O1^2^ | 61.29(4) |  | S1^13^ | S2B | Sr1^8^ | 106.30(5) |
| O2^6^ | Sr2 | O1^8^ | 128.61(4) |  | S1^7^ | S2B | Sr1^2^ | 106.30(5) |
| O2 | Sr2 | O1^8^ | 137.51(4) |  | S1^7^ | S2B | Sr1^8^ | 106.30(5) |
| O2^1^ | Sr2 | O1^8^ | 61.29(4) |  | S1^13^ | S2B | Sr1^2^ | 106.30(5) |
| O2 | Sr2 | O2^9^ | 74.67(6) |  | S1^13^ | S2B | Sr2^12^ | 96.15(12) |
| O2^6^ | Sr2 | O2^1^ | 74.13(5) |  | S1^7^ | S2B | Sr2^12^ | 96.15(12) |
| O2^9^ | Sr2 | O2^6^ | 142.370(15) |  | S1^13^ | S2B | Sr2 | 89.41(8) |
| Atom1 | Atom2 | Atom3 | Angle(*˚*) |  | Atom1 | Atom2 | Atom3 | Angle(*˚*) |
| O2 | Sr2 | O2^1^ | 142.370(15) |  | S1^7^ | S2B | Sr2 | 89.41(8) |
| O2^9^ | Sr2 | O2^1^ | 93.49(4) |  | S1^13^ | S2B | S1^7^ | 0.00(3) |
| O2 | Sr2 | O2^6^ | 93.49(4) |  |  |  |  |  |

^1^1-X,-1/2+Y,3/2-Z; ^2^1-X,1-Y,1-Z; ^3^+X,1/2-Y,1-Z; ^4^-X,1-Y,1-Z; ^5^-X,-1/2+Y,+Z; ^6^1-X,-1/2+Y,+Z; ^7^2-X,

-1/2+Y,3/2-Z; ^8^1-X,1-Y,1/2+Z; ^9^+X,+Y,3/2-Z; ^10^1-X,1/2+Y,3/2-Z; ^11^+X,3/2-Y,1-Z; ^12^2-X,1/2+Y,3/2-Z; ^13^2-X,-1/2+Y,+Z

**Table S6.** Atomic Occupancy for Sr_4_(VO_4_)_2_S_3_.

| Atom | Occupancy |  | Atom | Occupancy |  | Atom | Occupancy |
| --- | --- | --- | --- | --- | --- | --- | --- |
| S1 | 0.5 |  | S2A | 0.5 |  | S2B | 0.5 |

**Table S7.** Comparison of the birefringence and stable temperature of Sr_4_(VO_4_)_2_S_3_ with those of all commercial birefringent crystals, recently reported famous birefringent crystals and optical crystals composed of tetrahedral structural units.

| **unit** | **Compounds** | **Space group** | **Birefringence (nm)** | **Stable temperature (K)** | **Band gap (eV)** | **References** |
| --- | --- | --- | --- | --- | --- | --- |
| SO_4_^2-^ | Cs_2_Mg_3_(SO_4_)_4_ | *P*2_1_2_1_2_1_ | 0.0016@534 | 1057 |  | ^23^ |
|  | Li_5_Cs(SO_4_)_3_ | *P*2_1_/*c* | 0.0046@1064 | 913 | 6.62 | ^24^ |
|  | (NH_4_)_2_SO_4_ | *Pnam* | 0.011@546 | 508 | 4.90 | ^25^ |
|  | NaHSO_4_·H_2_O | *Cc* | 0.042@546 | 393 | 5.13 | ^26^ |
|  | NaRbY_2_(SO_4_)_4_ | *C*2/*c* | 0.045@550 | 949 | 5.71 | ^27^ |
|  | NaK_5_La_2_(SO_4_)_6_ | *C*2/*m* | 0.0255@550 | 723 | 5.3 | ^28^ |
|  | NH_4_NaLi_2_(SO_4_)_2_ | *C*2 | 0.009@550 | 516 |  | ^29^ |
|  | Li_8_NaRb_3_(SO_4_)_6_·2H_2_O | *C*2 | 0.021@546 | 438 | 4.86 | ^30^ |
|  | CeF_2_(SO_4_) | *Pna*2_1_ | 0.36@546 | 678 | 2.71 | ^31^ |
| PO_4_^3-^ | KMg(H_2_O)PO_4_ | *Pmn*2_1_ | 0.017@1064 | 453 | 6.20 | ^32^ |
|  | Mg_2_PO_4_Cl | *Pna*2_1_ | 0.046@1064 | 1170 |  | ^33^ |
|  | LiCs_2_PO_4_ | *Cmc*2_1_ | 0.01@546 |  | 7.02 | ^34^ |
|  | BaZn(PO_4_)F | *Pna*2_1_ | 0.011@1064 | 873 | 6.64 | ^35^ |
|  | BPO_4_ | *I*-4 | 0.006@546 | 1673 |  | ^36^ |
|  | LaPO_4_ | *I*4_1_/*amd* | 0.099@515 |  | 6.43 | ^37^ |
|  | YPO_4_ | *I*4_1_/*amd* | 0.1413@515 |  | 5.86 | ^37^ |
|  | ScPO_4_ | *I*4_1_/*amd* | 0.2232@515 |  | 4.70 | ^37^ |
| SiO_4_^4-^ | Li_2_BeSiO_4_ | *Pmn*2_1_ | 0.016@590 | 1129 | 7.05 | ^38^ |
|  | Na_2_BeSiO_4_ | *Pbc*2_1_ | 0.0086@590 | 1429 | 6.75 | ^38^ |
| VO_4_^3-^ | **Sr_4_(VO_4_)_2_S_3_** | ***Pbcm*** | **0.52@550** | **957** | **2.29** | **This work** |
|  | Li_3_VO_4_ | *Pmn*2_1_ | 0.021@546 | 1273 | 4.27 | ^39^ |
|  | LiRb_2_VO_4_ | *Cmc*2_1_ | 0.025@546 |  | 4.07 | ^40^ |
|  | LiCs_2_VO_4_ | *Cmc*2_1_ | 0.028@546 |  | 3.95 | ^40^ |
| SO_3_F^-^ | KSO_3_F | *Pnma* | 0.024@546 | 623 | 5.06 | ^25^ |
|  | CsSO_3_F | *P*2_1_/*c* | 0.029@1064 |  | 5.16 | ^25^ |
|  | LiSO_3_F | *Pna*2_1_ | 0.055@1064 |  | 5.82 | ^25^ |
|  | NH_4_SO_3_F | *Pnma* | 0.043@1064 |  | 5.02 | ^25^ |
|  | YSO_4_F·H_2_O | *P*2_1_/*n* | 0.0346@1064 | 573 | 5.90 | ^41^ |
| PO_3_F^2-^ | (NH_4_)_3_[PO_3_F][BF_4_] | *P*2_1_/*m* | 0.012@1064 | 423 | 5.02 | ^42^ |
|  | Na_2_PO_3_F | *C*2/*c* | 0.039@1064 | 898 | 4.19 | ^43^ |
|  | NaNH_4_PO_3_F·H_2_O | *Pn* | 0.035@532 | 373 | 7.02 | ^43^ |
|  | (NH_4_)_2_PO_3_F | *Pna*2_1_ | 0.035@532 |  | 7.59 | ^43^ |
|  | KHPO_3_F | *P*2_1_ | 0.028@532 |  | 6.92 | ^43^ |
|  | Cd(NH_4_)_2_(PO_3_F)_2_·2H_2_O | *Cmc*2_1_ | 0.026@546 | 410 | 4.01 | ^44^ |
|  | [C(NH_2_)_3_]_2_PO_3_F | *Cm* | 0.039@532 |  | 6.55 | ^45^ |
| NH_2_SO_3_^-^ | K_2_Ca(NH_2_SO_3_)_4_ | *P*-1 | 0.035@532 | 478 | 5.566 | ^46^ |
|  | Rb_2_Ca(NH_2_SO_3_)_4_ | *P*-1 | 0.036@532 | 478 | 5.532 | ^46^ |
|  | Cs_2_Mg(NH_2_SO_3_)_4_·4H_2_O | *Cm* | 0.054@546 | 338 | 4.949 | ^46^ |
|  | Sr(NH_2_SO_3_)_2_ | *Pc* | 0.056@589.3 | 551 | 7.32 | ^47^ |
|  | Ba(NH_2_SO_3_)_2_ | *Pna*2_1_ | 0.03@532 | 544 | 7.29 | ^47^ |
| PO_3_NH_3_^-^ | NaPO_3_NH_3_ | *P*6_3_ | 0.062@546.1 | 533 | >6.5 | ^48^ |
| SO_3_S^2-^ | (NH_4_)_2_S_2_O_3_ | *C*2 | 0.077@546 | 443 | 4.44 | ^49^ |
| PO_3_S^3-^ | Na_3_PO_3_S | *R*3*c* | 0.088@1064 |  | 5.29 | ^50^ |
|  | KH_2_PO_3_S | *Cm* | 0.129@1064 |  | 6.13 | ^50^ |
| [BO_2_]_∞_ | Ca(BO_2_)_2_ | *Pbcn* | 0.123@1064 | 1425 | 7.34 | ^51^ |
|  | LiBO_2_ | *P*2_1_/*c* | 0.135@1064 | 1118 | 7.56 | ^52^ |
| [BO_3_] | BaAl_2_B_2_O_7_ | *R*32 | 0.063@589.3 |  |  | ^53^ |
|  | LaBO_3_ | *Pnma* | 0.093@546 |  | 5.23 | ^54^ |
|  | Ca_3_(BO_3_)_2_ | *R*3*c* | 0.098@546 |  | 6.89 | ^55^ |
|  | Li_3_La_2_(BO_3_)_3_ | *P*2_1_/*c* | 0.078@1064 |  | 5.22 | ^56^ |
|  | Li_2_RbY_4_B_5_O_15_ | *P*2/*n* | 0.104@532 | 1124 | 4.65 | ^57^ |
|  | Li_2_CsY_4_B_5_O_15_ | *P*2/*n* | 0.103@532 | 1087 | 4.87 | ^57^ |
| [B_2_O_5_] | Li_2_Na_2_B_2_O_5_ | *Cmcm* | 0.095@532 | 882 |  | ^58^ |
| [B_3_O_6_] | Ba_2_Mg(B_3_O_6_)_2_ | *R*-3 | 0.1042@546.1 |  |  | ^59^ |
|  | Ba_2_Ca(B_3_O_6_)_2_ | *R*-3 | 0.1157@587.6 | 1389 |  | ^60^ |
|  | Ba_2_Cd(B_3_O_6_)_2_ | *R*-3 | 0.0875@270 | 1255 | 4.59 | ^61^ |
|  | RbBaYB_6_O_12_ | *R*-3 | 0.100@589 | 1266 | 5.11 | ^62^ |
|  | CsBaYB_6_O_12_ | *R*-3 | 0.100@589 | 1189 | 5.06 | ^62^ |
|  | Na_3_Ba_2_(B_3_O_6_)_2_F | *P*6_3_/*m* | 0.111@532 | 1093 | 4.676 | ^63^ |
| [BO_2_F_2_] | Na_3_B_7_O_11_F_2_ | *Pnma* | 0.083@193 |  | 7.69 | ^64^ |
| [B_4_O_6_F]_∞_ | NaB_4_O_6_F | *C*2 | 0.12@1064 | 807 | 7.57 | ^65^ |
|  | RbB_4_O_6_F | *Pna*2_1_ | 0.102@546 | 725 |  | ^66^ |
|  | CsB_4_O_6_F | *Pna*2_1_ | 0.122@514 | 882 |  | ^67^ |
|  | NH_4_B_4_O_6_F | *Pna*2_1_ | 0.126@514 | 573 | 7.87 | ^68^ |
|  | BaB_8_O_12_F_2_ | *R*-3*c* | 0.116@1064 | 890 | 6.85 | ^69^ |
| [B_4_O_6_F_2_]_∞_ | CaB_4_O_6_F_2_ | *P*-1 | 0.091@1064 |  | 6.06 | ^70^ |
|  | SrB_4_O_6_F_2_ | *P*-1 | 0.089@1064 |  | 6.14 | ^70^ |
|  | BaB_4_O_6_F_2_ | *P*2_1_/*n* | 0.085@1064 |  | 6.76 | ^70^ |
| [B_5_O_7_F_3_]_∞_ | MgB_5_O_7_F_3_ | *Cmc*2_1_ | 0.07@1064 | 913 | 6.21 | ^71^ |
|  | CaB_5_O_7_F_3_ | *Cmc*2_1_ | 0.07@1064 | 913 | 7.05 | ^72^ |
|  | SrB_5_O_7_F_3_ | *Cmc*2_1_ | 0.073@532 |  | 6.89 | ^73^ |
|  | PbB_5_O_7_F_3_ | *Cmc*2_1_ | 0.12@1064 | 753 | 5.56 | ^74^ |
| [B_6_O_9_F_2_]_∞_ | Li_2_B_6_O_9_F_2_ | *Pnn*2 | 0.067@1064 | 523 | 7.60 | ^75^ |
| [GeS_3_(S_2_)] | K_2_BaGeS_3_(S_2_) | *P*2_1_/*c* | 0.147@546 |  |  | ^76^ |
| [Ge_2_S_5_(S_2_)] | Li_2_Cs_4_Ge_2_S_5_(S_2_)Cl_2_ | *C*222_1_ | 0.13@1064 |  | 2.92 | ^77^ |
| [Nb_2_S_8_(S_2_)_2_] | Ga_2_NbS_4_(S_2_) | *Cm* | 0.29@2000 | 1013 | 1.80 | ^78^ |
| S_2_^2-^ | Ba_4_(S_2_)ZnGa_4_S_10_ | *I*222 | 0.053@1064 |  | 3.39 | ^79^ |
| S_3_^2-^ | Sr_6_(Sb_6_S_14_)(S_3_) | *P*2_1_2_1_2_1_ | 0.29@2.1 |  | 1.92 | ^80^ |
| S_2_^2-^、S_3_^2-^ | Na_4_Ba_3_(S_2_)_4_S_3_ | *Fdd*2 | 0.37@1064 |  | 2.30 | ^81^ |
| C_2_O_4_^2-^ | (NH_4_)_2_C_2_O_4_·H_2_O | *P*2_1_2_1_2_1_ | 0.248@546 |  | 3.39 | ^82^ |
| C_5_O_5_^2-^ | Na_2_C_5_O_5_·2H_2_O | *C*2/*c* | 1.062@546 | 319 | 2.67 | ^83^ |
|  | Na_2_C_5_O_5_·3H_2_O | *P*2_1_/*c* | 0.893@546 | 326 | 2.55 | ^83^ |
| [H_2_C_6_N_7_O_3_]^-^ | Ba(H_2_C_6_N_7_O_3_)_2_·8H_2_O | *Fdd*2 | 0.24@550 | 350 | 4.10 | ^84^ |
|  | Cd(H_2_C_6_N_7_O_3_)_2_·8H_2_O | *P*2_1_ | 0.6@550 | 358 | 4.00 | ^85^ |
| [H_2_C_6_N_9_]^-^ | Cs(H_2_C_6_N_9_)·H_2_O | *P*-1 | 0.55@550 | 400 | 4.12 | ^86^ |
| [C_9_N_13_]^3-^ | Li_3_(C_9_N_13_)·6H_2_O | *P*6_3_/*m* | 1.031@546 | 424 | 3.62 | ^87^ |
| [HC_3_N_3_S_3_]^2-^ | Cs_3_Cl(HC_3_N_3_S_3_) | *Pmc*2_1_ | 0.52@550 | 570 | 3.34 | ^88^ |
| [C_6_H_4_NO_2_]^-^ | I^+^(C_6_H_4_NO_2_)^-^ | *P*2_1_/*m* | 0.778@550 | 567 | 3.08 | ^86^ |
| [C_7_H_3_NO_4_]^2-^ | [Be_2_(*μ*-OH)_2_(C_7_H_3_NO_4_)(H_2_O)]·H_2_O | *P*2_1_ | 0.6@550 | 433 | 4.75 | ^89^ |
| [C_12_H_8_N_2_] | (C_12_H_8_N_2_)SbF_2_(H_2_PO_3_) | *P*-1 | 0.79@546 | 423 | 3.2 | ^90^ |
|  | (C_12_H_8_N_2_)SbF_3_ | *P*2_1_/*c* | 0.43@546 | 483 | 3.45 | ^90^ |
| [C_6_H_4_NO_2_]^2-^ | (C_6_H_4_NO_2_)_2_SbF | *P*2_1_/*n* | 0.38@546 | 493 | 3.71 | ^91^ |
| [C_10_H_6_NO_2_]^2-^ | (C_10_H_6_NO_2_)_2_SbF | *P*-1 | 0.87@546 | 533 | 3.35 | ^91^ |
| [C_2_N_4_OH_7_]^+^ | (C_2_N_4_OH_7_)(H_2_PO_3_) | *Cc* | 0.19@589.3 | 455 | 4.96 | ^92^ |
|  | (C_2_N_4_OH_7_)(NH_2_SO_3_) | *P*1 | 0.225@1064 | 478 | 4.97 | ^93^ |
| 4-HPy | (4-HPy)(H_2_PO_4_) | *P*2_1_2_1_2_1_ | 0.25@1064 | 439 | 4.69 | ^94^ |
|  | (4-HPy)(CH_3_SO_3_) | *Pna*2_1_ | 0.216@546 | 346 | 4.64 | ^95^ |
|  | (4-HPy)(NO_3_) | *P*1 | 0.494@546 | 479 | 3.73 | ^96^ |
|  | (4-HPy)_2_ZnCl_2_ | *C*2/*c* | 0.482@546 | 493 | 4.29 | ^97^ |
|  | (4-HPy)_2_ZnCl_4_ | *C*2/*c* | 0.193@546 | 453 | 4.52 | ^97^ |
|  | (4-HPy)(Sb_2_OF_4_) | *Cm* | 0.513@546 | 508 | 4.59 | ^98^ |
|  | Na_2_(4-HPyH)_2_(PTS)·H_2_O | *P*-1 | 0.811@546 | 403 | 3.05 | ^99^ |
| [HPyBrB(C_2_O_4_)]^+^ | [HPyBrB(C_2_O_4_)](OH) | *P*2_1_/*c* | 0.194@546 | 423 |  | ^100^ |
| [HPyClB(OH)_2_]^+^ | [HPyClB(OH)_2_](NO_3_) | *P*2_1_/*c* | 0.533@546 | 383 | 4.07 | ^100^ |
| 4-AP | (4-AP)(Sb_2_F_7_) | *Pn* | 0.134@546 | 473 | 4.51 | ^98^ |
|  | (4-AP)(3-PySO_3_) | *P*2_1_/*c* | 0.296@546 | 490 | 4.00 | ^101^ |
| 2-AP | (2-AP)(H_2_PO_3_) | *P*2_1_ | 0.225@546 | 439 | 3.27 | ^102^ |
|  | (2-AP)(NH_2_SO_3_) | *Cm* | 0.233@546 | 423 | 2.90 | ^103^ |
| 3-PySO_3_ | K(3-PySO_3_) | *Pna*2_1_ | 0.306@546 |  | 4.26 | ^104^ |
| MEL | (MEL)(PbBr_4_) | *P*2_1_/*c* | 0.322@550 | 550 | 3.13 | ^105^ |
|  | (MEL)(SO_3_CF_3_) | *P*-1 | 0.380@546 | 474 | 4.78 | ^106^ |
|  | C_3_H_8_N_6_I_6_·3H_2_O | *P*2_1_/*n* | 2.8 |  | 1.84 | ^107^ |
| [HC_3_N_3_O_3_]^2-^ | KLi(HC_3_N_3_O_3_)·2H_2_O | *Pna*2_1_ | 0.186@514 | 398 | 5.23 | ^108^ |
|  | Rb_2_(HC_3_N_3_O_3_) | *Cmcm* | 0.4@532 | 665 | 4.96 | ^109^ |
| Commercial | MgF_2_ | *P*4_2_/*mnm* | 0.012@532 | 1521 | 11.8 | ^110^ |
|  | LiNbO_3_ | *R*3*c* | 0.074@546 | 1513 | 3.80 | ^111^ |
|  | *α*-BaB_2_O_4_ | *R*-3*c* | 0.122@546 | 1333 | 6.56 | ^112^ |
|  | CaCO_3_ | *R*-3*c* | 0.172@532 | 1098 | 6.00 | ^113^ |
|  | YVO_4_ | *I*4_1_/*amd* | 0.235@546 | 2098 | 3.70 | ^114^ |
|  | TiO_2_ | *P*4_2_/*mnm* | 0.256@546 | 2113 | 3.20 | ^115^ |

# Table S8. Static polarizability (*α*) of (VO_4_)^3-^ and (S_3_)^2-^ (a.u.).

| Unit | *XX* | *XY* | *YY* | *XZ* | *YZ* | *ZZ* |
| --- | --- | --- | --- | --- | --- | --- |
| (S_3_)^2-^ | 237.9 | 0 | 127.5 | 0 | 0 | 145.1 |
| (VO_4_)^3-^ | 85.6 | 0 | 85.5 | 0 | 0 | 85.5 |

# References

1. Dolomanov, O.V., Bourhis, L.J., Gildea, R.J., Howard, J.A.K. & Puschmann, H. OLEX2: a Complete Structure Solution, Refinement and Analysis Program. *J. Appl. Crystallogr.* **42**, 339-341 (2009).

2. Sheldrick, G.M. A Short History of SHELX. *Acta Crystallogr. Sect. A* **64**, 112-122 (2008).

3. Sheldrick, G.M. Crystal Structure Refinement with SHELXL. *Acta Crystallogr. Sect. C-Struct. Chem.* **71**, 3-8 (2015).

4. Spek, A.L. Single-Crystal Structure Validation with the Program PLATON. *J. Appl. Crystallogr.* **36**, 7-13 (2003).

5. Tauc, J. Absorption Edge and Internal Electric Fields in Amorphous Semiconductors. *Mater. Res. Bull.* **5**, 721-729 (1970).

6. Cao, L.L. et al. A Microcrystal Method for the Measurement of Birefringence. *Crystengcomm* **22**, 1956-1961 (2020).

7. Sorensen, B.E. A Revised Michel-Levy Interference Colour Chart Based on First-Principles Calculations. *Eur. J. Mineral.* **25**, 1041-1041 (2013).

8. Clark, S.J. et al. First Principles Methods Using CASTEP. *Z. Kristallogr.* **220**, 567-570 (2005).

9. Payne, M.C., Teter, M.P., Allan, D.C., Arias, T.A. & Joannopoulos, J.D. Iterative Minimization Techniques for ab Initio Total-energy Calculations: Molecular Dynamics and Conjugate Gradients. *Rev. Mod. Phys.* **64**, 1045-1097 (1992).

10. Kohn, W. & Sham, L.J. Self-Consistent Equations Including Exchange and Correlation Effects. *Phys. Rev.* **140**, 1133-& (1965).

11. Perdew, J.P., Burke, K. & Ernzerhof, M. Generalized Gradient Approximation Made Simple. *Phys. Rev. Lett.* **78**, 1396-1396 (1997).

12. Pfrommer, B.G., Cote, M., Louie, S.G. & Cohen, M.L. Relaxation of Crystals with the Quasi-Newton Method. *J. Comput. Phys.* **131**, 233-240 (1997).

13. Rappe, A.M., Rabe, K.M., Kaxiras, E. & Joannopoulos, J.D. Optimized Pseudopotentials. *Phys. Rev. B* **41**, 1227-1230 (1990).

14. Monkhorst, H.J. & Pack, J.D. Special Points for Brillouin-Zone Integrations. *Phys. Rev. B* **13**, 5188-5192 (1976).

15. Frisch, M. J. et al. *Gaussian 16*, Revision B.01, Gaussian, Inc., Wallingford CT (2016).

16. Lu, T. & Chen, F. Multiwfn: A Multifunctional Wavefunction Analyzer. *J. Comput. Chem.* **33**, 580-592 (2012).

17. Humphrey, W., Dalke, A. & Schulten, K. VMD: Visual Molecular Dynamics. *J. Mol. Graph.* **14**, 33-38 (1996).

18. Alparone, A. Linear and Nonlinear Optical Properties of Nucleic Acid Bases. *Chem. Phys.* **410**, 90-98 (2013).

19. Jiang, X. et al. The Role of Dipole Moment in Determining the Nonlinear Optical Behavior of Materials: ab Initio Studies on Quaternary Molybdenum Tellurite Crystals. *J. Mater. Chem. C* **2**, 530-537 (2014).

20. Brown, I.D. The Chemical Bond in Inorganic Chemistry: the Bond Valence Model. *Oxford University Press: Oxford, UK* (2016).

21. Maroulis, G. Hyperpolarizability of H_2_O. *J. Chem. Phys.* **94**, 1182-1190 (1991).

22. Brese, N. & O'keeffe, M. Bond-Valence Parameters for Solids. *Struct. Sci.* **47**, 192-197 (1991).

23. Wang, M., Wei, D., Liang, L., Yan, X. & Lv, K. Centrosymmetric Rb_2_Mg_3_(SO_4_)_4_ and Non-Centrosymmetric Cs_2_Mg_3_(SO_4_)_4_ with a Phase-Matching Nonlinear Optical Response. *Inorg. Chem. Commun.* **107**, 107486 (2019).

24. Yan, Y.C., Chen, Y.N., Jiang, B., Jing, Q. & Zhang, J. Li_5_Cs(SO_4_)_3_: a Potential Zero-Order Wave Plate Material with Short Deep-Ultraviolet Cutoff Edge. *New J. Chem.* **45**, 19883-19888 (2021).

25. Jin, W. et al. Fluorine‐Driven Enhancement of Birefringence in the Fluorooxosulfate: a Deep Evaluation from a Joint Experimental and Computational Study. *Adv. Sci.* **8**, 2003594 (2021).

26. Li, H. et al. NaHSO_4_·H_2_O: A Promising Deep-Ultraviolet Nonlinear-Optical Bisulfate with Large Birefringence and a Second-Harmonic-Generation Effect. *Inorg. Chem.* **61**, 19673-19677 (2022).

27. Zhao, Y.Q. et al. Deep-Ultraviolet Bialkali-Rare Earth Metal Anhydrous Sulfate Birefringent Crystal. *Inorg. Chem.* **63**, 11187-11193 (2024).

28. Liu, L.H., Yuan, F.F., Zhang, L.Z., Huang, Y.S. & Lin, Z.B. NaK_5_La_2_(SO_4_)_6_: Enhanced Birefringence of Multiple-Alkali Metal Sulfate Systems via Rare Earth Metal-Centered Polyhedra. *Inorg. Chem.* **63**, 14721-14726 (2024).

29. Li, Y.Q. et al. Two Non-π-Conjugated Deep-UV Nonlinear Optical Sulfates. *J. Am. Chem. Soc.* **141**, 3833-3837 (2019).

30. Li, Y.Q. et al. Li_8_NaRb_3_(SO_4_)_6_•2H_2_O as a New Sulfate Deep-Ultraviolet Nonlinear Optical Material. *J. Mater. Chem. C* **6**, 12240-12244 (2018).

31. Wu, C. et al. Large Second-Harmonic Response and Giant Birefringence of CeF_2_(SO_4_) Induced by Highly Polarizable Polyhedra. *J. Am. Chem. Soc.* **143**, 4138-4142 (2021).

32. Bai, Z.Y. et al. KMg(H_2_O)PO_4_: A Deep-Ultraviolet Transparent Nonlinear Optical Material Derived from KTiOPO_4_. *Chem. Mater.* **31**, 9540-9545 (2019).

33. Zhang, J.X. et al. Screening Strategy Identifies an Overlooked Deep‐Ultraviolet Transparent Nonlinear Optical Crystal. *Angew. Chem. Int. Ed.* **63**, e202413276 (2024).

34. Li, L. et al. A New Deep-Ultraviolet Transparent Orthophosphate LiCs_2_PO_4_ with Large Second Harmonic Generation Response. *J. Am. Chem. Soc.* **138**, 9101-9104 (2016).

35. Tang, X.Y. et al. Rational Design and Synthesis of a Deep-Ultraviolet Nonlinear Optical Fluorinated Orthophosphate: BaZn(PO_4_)F. *Inorg. Chem.* **58**, 4508-4514 (2019).

36. Zhang, X. et al. Optical Properties of the Vacuum-Ultraviolet Nonlinear Optical Crystal-BPO_4_. *J. Opt. Soc. Am. B-Opt. Phys.* **28**, 2236-2239 (2011).

37. Tuerhong, N. et al. The Enhanced Bandgap and Birefringence of Rare-Earth Phosphates XPO_4_ (X = Sc, Y, La, and Lu): A First-Principles Investigation. *Phys. Chem. Chem. Phys.* **26**, 15751-15757 (2024).

38. Chen, Y.G. et al. Two α-SiO_2_-Related Deep-Ultraviolet Phase-Matchable Optical Nonlinear Beryllium Silicate Crystals Na_2_BeSiO_4_ and Li_2_BeSiO_4_ with Enhanced SHG Effect. *Small* **21**, 2408360 (2025).

39. Chen, Z.H. et al. Li_3_VO_4_: A Promising Mid -Infrared Nonlinear Optical Material with Large Laser Damage Threshold. *Cryst. Growth Des.* **17**, 2792-2800 (2017).

40. Su, X. et al. Intense d-p Hybridization Induced a Vast SHG Response Disparity between Tetrahedral Vanadates and Arsenates. *J. Phys. Chem. C* **124**, 24949-24956 (2020).

41. Jiao, D.X. et al. YSO_4_F·H_2_O: A Deep-Ultraviolet Birefringent Rare-Earth Sulfate Fluoride with Enhanced Birefringence Induced by Fluorinated Y-Centered Polyhedra. *Inorg. Chem.* **62**, 17333-17340 (2023).

42. Qiu, H. et al. Tetrafluoroborate-Monofluorophosphate (NH_4_)_3_[PO_3_F][BF_4_]: First Member of Oxyfluoride with B–F and P–F Bonds. *ACS Org. Inorg. Au* **1**, 6-10 (2021).

43. Zhang, B.B. et al. Expanding Frontiers of Ultraviolet Nonlinear Optical Materials with Fluorophosphates. *Chem. Mater.* **30**, 5397-5403 (2018).

44. Yang, X.R., Liu, X., Deng, X.B., Chen, L. & Wu, L.M. Isomeric Cd(NH_4_)_2_(PO_3_F)_2_.2H_2_O: Solution Concentration-Driven Elimination of Antiparallel Dipole-Dipole Interaction Generating an SHG β-Phase. *Mater. Today Phys.* **31**, 8 (2023).

45. Xiong, L., Chen, J., Lu, J., Pan, C.Y. & Wu, L.M. Monofluorophosphates: A New Source of Deep-Ultraviolet Nonlinear Optical Materials. *Chem. Mater.* **30**, 7823-7830 (2018).

46. Wang, X. et al. Deep‐Ultraviolet Transparent Mixed Metal Sulfamates with Enhanced Nonlinear Optical Properties and Birefringence. *Angew. Chem. Int. Ed.* **136**, e202315434 (2024).

47. Hao, X. et al. M(NH_2_SO_3_)_2_ (M=Sr, Ba): Two Deep-Ultraviolet Transparent Sulfamates Exhibiting Strong Second Harmonic Generation Responses and Moderate Birefringence. *Angew. Chem. Int. Ed.* **60**, 7621-7625 (2021).

48. Wu, L.L. et al. Optimized Arrangement of Non-π-Conjugated PO_3_NH_3_ Units Leads to Enhanced Ultraviolet Optical Nonlinearity in NaPO_3_NH_3_. *Inorg. Chem. Front.* **11**, 1145-1152 (2024).

49. Ke, S.X., Fan, H.X., Lin, C.S., Ye, N. & Luo, M. Constructing Ultraviolet Nonlinear Optical Crystals with Large Second Harmonic Generation and Short Absorption Edges by Using Polar Tetrahedral S_2_O_3_ Groups. *Inorg. Chem. Front.* **10**, 2811-2817 (2023).

50. Zhang, X.Y., Kang, L., Gong, P.F., Lin, Z.S. & Wu, Y.C. Nonlinear Optical Oxythiophosphate Approaching the Good Balance with Wide Ultraviolet Transparency, Strong Second Harmonic Effect, and Large Birefringence. *Angew. Chem. Int. Ed.* **60**, 6386-6390 (2021).

51. Chen, X.L. et al. Designing an Excellent Deep-Ultraviolet Birefringent Material for Light Polarization. *J. Am. Chem. Soc.* **140**, 16311-16319 (2018).

52. Zhang, F. et al. An Excellent Deep-Ultraviolet Birefringent Material Based on [BO_2_]_∞_ Infinite Chains. *Light Sci. Appl.* **11**, 252 (2022).

53. Ye, N., Zeng, W., Wu, B. & Chen, C. Two New Nonlinear Optical Crystals: BaAl_2_B_2_O_7_ and K_2_Al_2_B_2_O_7_. *Proc. SPIE* **3556**, 21-23 (1998).

54. Sha, H.Y. et al. A New Rare-Earth Borate Birefringent Crystal with Quasi-Two-Dimensional [BO_3_] Layers. *J. Mater. Chem. C* **9**, 15886-15890 (2021).

55. Zhang, S.Y. et al. Growth of Birefringent Ca_3_(BO_3_)_2_ Crystals by the Czochralski Method. *J. Cryst. Growth* **252**, 246-250 (2003).

56. Shi, X.P. et al. Li_3_La_2_(BO_3_)_3_ and Li_1.75_Na_1.25_La_2_(BO_3_)_3_: A Great Enhancement in Birefringence Induced by Optimal Arrangement of π-Conjugated [BO_3_] Units. *Inorg. Chem.* **60**, 12565-12572 (2021).

57. Gao, M.G. et al. Inducing Large Birefringence by Enhancing Asymmetric Electron Distribution of Y-O Polyhedra. *Inorg. Chem. Front.* **9**, 1956-1963 (2022).

58. Zhang, M. et al. Rational Design via Synergistic Combination Leads to an Outstanding Deep-Ultraviolet Birefringent Li_2_Na_2_B_2_O_5_ Material with an Unvalued B_2_O_5_ Functional Gene. *J. Am. Chem. Soc.* **141**, 3258-3264 (2019).

59. Zhao, J., Ma, Y.Y. & Li, R.K. Characterization of Polarizer Made of the Deep-UV Birefringent Crystal Ba_2_Mg(B_3_O_6_)_2_. *Appl. Opt.* **54**, 9949-9953 (2015).

60. Jia, Z. et al. Top-Seeded Solution Growth and Optical Properties of Deep-UV Birefringent Crystal Ba_2_Ca(B_3_O_6_)_2_. *Cryst. Growth Des.* **17**, 558-562 (2017).

61. Dong, X.Y. et al. Ba_2_Cd(B_3_O_6_)_2_: A Congruent-Melting Compound with Isolated B_3_O_6_ Units. *Z. Anorg. Allg. Chem.* **639**, 988-993 (2013).

62. Chen, X.L. et al. MBaYB_6_O_12_ (M = Rb, Cs): two New Rare-Earth Borates With Large Birefringence and Short Ultraviolet Cutoff Edges. *Dalton Trans.* **47**, 750-757 (2018).

63. Zhang, H. et al. Na_3_Ba_2_(B_3_O_6_)_2_F: Next Generation of Deep-Ultraviolet Birefringent Materials. *Cryst. Growth Des.* **15**, 523-529 (2015).

64. Tang, C. et al. Na_3_B_7_O_11_F_2_: a New Sodium-Rich Fluorooxoborate with a Unique [B_14_O_24_F_4_] Ring and a Short Ultraviolet Absorption Edge. *Dalton Trans.* **48**, 21-24 (2019).

65. Zhang, Z.Z., Wang, Y., Zhang, B.B., Yang, Z.H. & Pan, S.L. Polar Fluorooxoborate, NaB_4_O_6_F: A Promising Material for Ionic Conduction and Nonlinear Optics. *Angew. Chem. Int. Ed.* **57**, 6577-6581 (2018).

66. Cheng, H., Pan, S. & Yang, Z. Non-Equivalent Terminating Roles of Hydroxyl and Fluorine in Hydroxy/Fluorooxo-Borate Optical Functional Materials. *Sci. China. Chem.* **68**, 4761-4770 (2025).

67. Wang, X. et al. CsB_4_O_6_F: a Congruent‐Melting Deep‐Ultraviolet Nonlinear Optical Material by Combining Superior Functional Units. *Angew. Chem. Int. Ed.* **129**, 14307-14311 (2017).

68. Zhang, Q. et al. Review on Birefringence in Borates Based on Birefringence-Active Functional Groups and Arrangements. *Sci. China. Chem.* **67**, 2155-2170 (2024).

69. Zhang, Z., Wang, Y., Li, H., Yang, Z. & Pan, S. BaB_8_O_12_F_2_: A Promising Deep-UV Birefringent Material. *Inorg. Chem. Front.* **6**, 546-549 (2019).

70. Zhang, Z., Wang, Y., Zhang, B., Yang, Z. & Pan, S. Designing Deep‐UV Birefringent Crystals by Cation Regulation. *Chem.–A Eur. J.* **24**, 11267-11272 (2018).

71. Xia, M. et al. Discovery of First Magnesium Fluorooxoborate with Stable Fluorine Terminated Framework for Deep‐UV Nonlinear Optical Application. *Angew. Chem. Int. Ed.* **133**, 14771-14777 (2021).

72. Zhang, Z., Wang, Y., Zhang, B., Yang, Z. & Pan, S. CaB_5_O_7_F_3_: A Beryllium-Free Alkaline-Earth Fluorooxoborate Exhibiting Excellent Nonlinear Optical Performances. *Inorg. Chem.* **57**, 4820-4823 (2018).

73. Mutailipu, M. et al. SrB_5_O_7_F_3_ Functionalized with [B_5_O_9_F_3_]^6-^ Chromophores: Accelerating the Rational Design of Deep‐Ultraviolet Nonlinear Optical Materials. *Angew. Chem. Int. Ed.* **130**, 6203-6207 (2018).

74. Han, S., Mutailipu, M., Tudi, A., Yang, Z. & Pan, S. PbB_5_O_7_F_3_: A High-Performing Short-Wavelength Nonlinear Optical Material. *Chem. Mater.* **32**, 2172-2179 (2020).

75. Li, X. et al. LiNaB_6_O_9_F_2_: A Promising UV NLO Crystal Having Fluorine-Directed Optimal Performances and Double Interpenetrating ^3^[B_6_O_9_F_2_]_∞_ Networks. *Adv. Opt. Mater.* **11**, 2202195 (2023).

76. Xie, W. et al. Improved Birefringence Activated by Tetrahedra Decorated with a Single Linear Unit. *Angew. Chem. Int. Ed.* **135**, e202307895 (2023).

77. Li, G.M., Wu, K., Huang, Y., Yang, Z.H. & Pan, S. Ge_2_S_5_(S_2_)^4-^, A NLO-Active Unit Leading to an Asymmetric Structure Discovered in Li_2_Cs_4_Ge_2_S_5_(S_2_)Cl_2_: An Experimental and Theoretical Study. *Chem.–A Eur. J.* **25**, 5440-5444 (2019).

78. Zhao, W.L., Wang, R.X., Zhao, S., Wu, L.M. & Chen, L. NbGa_2_S_4_(S_2_): A Rare Linear [S_2_]^2-^ Coordination to Niobium Resulting in Simultaneously Large SHG Effect and Giant Birefringence. *Angew. Chem. Int. Ed.* **64**, e202509238 (2025).

79. Ding, K. et al. Ba_4_(S_2_)ZnGa_4_S_10_: Design of an Unprecedented Infrared Nonlinear Salt-Inclusion Chalcogenide with Disulfide-Bonds. *Small* **19**, 2302819 (2023).

80. Xu, Q.-T., Yao, W.-D., Li, X.-H. & Guo, S.-P. Investigation of the Second-Order Nonlinear Optical Property of Sr_6_Sb_6_S_17_. *J. Solid State Chem.* **295**, 121915 (2021).

81. Dodge, M.J. Refractive Properties of Magnesium Fluoride. *J. Opt. Soc. Am. A.* **69**, 1460-1460 (1979).

82. Tong, T.H., Zhang, W.Y., Yang, Z.H. & Pan, S.L. Series of Crystals with Giant Optical Anisotropy: A Targeted Strategic Research. *Angew. Chem. Int. Ed.* **60**, 1332-1338 (2021).

83. Lu, J.C., Li, Y. & Ok, K.M. Five-Membered [C_5_O_5_]^2-^ Rings as Birefringence-Active Genes for Ultrabirefringent Crystals. *J. Am. Chem. Soc.* **147**, 35081-35089 (2025).

84. Li, Y. et al. A High‐Performance Nonlinear Optical Crystal with a Building Block Containing Expanded π‐Delocalization. *Angew. Chem. Int. Ed.* **62**, e202215145 (2023).

85. Li, Y. et al. A Hydrogen Bonded Supramolecular Framework Birefringent Crystal. *Angew. Chem. Int. Ed.* **135**, e202304498 (2023).

86. Li, Y.Q. et al. An Optically Anisotropic Crystal with Large Birefringence Arising from Cooperative π Orbitals. *Angew. Chem. Int. Ed.* **61**, e202208811 (2022).

87. Dou, D.Y., Wei, C., Zhang, B.B., Yang, D.Q. & Wang, Y. Ultra-High Optical Anisotropy with UV Transmission Achieved by Rational Arrangement of Extended π-Conjugated Groups. *Angew. Chem. Int. Ed.* **64**, e202504761 (2025).

88. Li, M.J. et al. A Hybrid Antiperovskite with Strong Linear and Second-Order Nonlinear Optical Responses. *Angew. Chem. Int. Ed.* **61**, e202211151 (2022).

89. Guo, P.H. et al. Giant Birefringence and Solar-Blind Ultraviolet Transmission by Coordination-Induced Assembly of π-Conjugated Pyridine-Carboxylate. *Angew. Chem. Int. Ed.* **64**, e202508997 (2025).

90. Zhang, P. et al. Double Sb-N Coordination Enables Record-High Birefringence in UV-Transparent Crystals. *Angew. Chem. Int. Ed.* **64**, e202424756 (2025).

91. Zhang, P. et al. Dual‐Sided Multidentate Coordination Strategy Enables Record Birefringence in UV‐Transparent Antimony‐Based Hybrid Crystals. *Angew. Chem. Int. Ed.* **64**, e202513511 (2025).

92. Xu, G., Bai, X., Yang, Z., Han, J. & Pan, S. π‐Conjugated Cations in Phosphates: A Pathway to Solar‐Blind UV Nonlinear Optical Crystals with Phase‐Matching. *Angew. Chem. Int. Ed.*, e202510363 (2025).

93. Wen, X. et al. [C_2_N_4_H_7_O][NH_2_SO_3_]: High-Performance Ultraviolet Nonlinear Optical Crystal with Ditrigon Coupled Guanylurea Group. *Angew. Chem. Int. Ed.* **64**, e202424153 (2025).

94. Lu, J. et al. Discovery of NLO Semiorganic (C_5_H_6_ON)^+^(H_2_PO_4_)^−^: Dipole Moment Modulation and Superior Synergy in Solar-Blind UV Region. *J. Am. Chem. Soc.* **143**, 3647-3654 (2021).

95. Zhang, Z.P. et al. Remarkable Second Harmonic Generation Response in (C_5_H_6_NO)^+^(CH_3_SO_3_)^-^: Unraveling the Role of Hydrogen Bond in Thermal Driven Nonlinear Optical Switch. *Angew. Chem. Int. Ed.* **63**, e202408551 (2024).

96. Yin, J.P. et al. Enhanced Coplanarity and Giant Birefringence in Hydroxypyridinium Nitrate via Hydrogen Bonding between Planar Donors and Planar Acceptors. *Angew. Chem. Int. Ed.* **64**, e202417579 (2025).

97. Li, Y. & Ok, K.M. Breaking Boundaries: Giant Ultraviolet Birefringence in Dimension-Reduced Zn-Based Crystals. *Angew. Chem. Int. Ed.* **63**, e202409336 (2024).

98. Qi, L. et al. Record Second-Harmonic Generation and Birefringence in an Ultraviolet Antimonate by Bond Engineering. *J. Am. Chem. Soc.* **146**, 9975-9983 (2024).

99. Jin, C. et al. Sulfonated Module Aggregation for Ultrahigh Birefringence in Aqueous-Processable Crystals. *J. Am. Chem. Soc.* (2025).

100. Li, J.J. et al. Breaking the Birefringence Barrier in B-O Crystals via B-C π-scaffolding. *J. Am. Chem. Soc.* **147**, 29864-29874 (2025).

101. Lu, J.C. & Ok, K.M. Synergistic Engineering of Ultraviolet Metal-Free Crystals with Exceptional Birefringence via Pyridine-Derived Dimers. *Chem. Sci.* **16**, 4703-4709 (2025).

102. Zhang, Z.P. et al. Driving Nonlinear Optical Activity with Dipolar 2-Aminopyrimidinium Cations in (C_4_H_6_N_3_)^+^(H_2_PO_3_)^-^. *Chem. Mater.* **34**, 1976-1984 (2022).

103. Dou, D.Y. et al. Rational Combination of π-Conjugated and Non-π-Conjugated Groups Achieving Strong Nonlinear Optical Response, Large Optical Anisotropy, and UV Light-Switchable Fluorescence. *Adv. Sci.* **11**, 2401325 (2024).

104. Bai, Z.Y. & Ok, K.M. Designing Sulfate Crystals with Strong Optical Anisotropy through π-Conjugated Tailoring. *Angew. Chem. Int. Ed.* **63**, e202315311 (2024).

105. Huang, W.Q. et al. A Hybrid Halide Perovskite Birefringent Crystal. *Angew. Chem. Int. Ed.* **61**, e202202746 (2022).

106. Jia, H. et al. Anionic Group Trimming Strategy: Oriented Fabrication of Sulfonate UV Birefringent Crystals with High Optical Anisotropy. *ACS Appl. Mater. Interfaces* **17**, 53808-53815 (2025).

107. Zhou, Y. et al. A Solution-Processable Natural Crystal with Giant Optical Anisotropy for Efficient Manipulation of Light Polarization. *Nat. Photonics* **18**, 922-927 (2024).

108. Lin, D.H., Luo, M., Lin, C.S., Xu, F. & Ye, N. KLi(HC_3_N_3_O_3_)•2H_2_O: Solvent-drop Grinding Method toward the Hydro-isocyanurate Nonlinear Optical Crystal. *J. Am. Chem. Soc.* **141**, 3390-3394 (2019).

109. Lu, J. et al. How To Maximize Birefringence and Nonlinearity of π-Conjugated Cyanurates. *J. Am. Chem. Soc.* **141**, 16151-16159 (2019).

110. Golota, A.F., Khubieva, Z.K., Khodos, M.Y. & Fotiev, A.A. Refractive Properties of Magnesium Fluoride. *Inorg. Mater.* **22**, 1827-1829 (1986).

111. Zelmon, D.E., Small, D.L. & Jundt, D. Infrared Corrected Sellmeier Coefficients for Congruently Grown Lithium Niobate and 5 mol.% Magnesium Oxide-Doped Lithium Niobate. *J. Opt. Soc. Am. B-Opt. Phys.* **14**, 3319-3322 (1997).

112. Zhou, G.Q. et al. Growth and Spectrum of a Novel Birefringent α-BaB_2_O_4_ Crystal. *J. Cryst. Growth* **191**, 517-519 (1998).

113. Ghosh, G. Dispersion-Equation Coefficients for the Refractive Index and Birefringence of Calcite and Quartz Crystals. *Opt. Commun.* **163**, 95-102 (1999).

114. Luo, H.T., Tkaczyk, T., Dereniak, E.L., Oka, K. & Sampson, R. High Birefringence of the Yttrium Vanadate Crystal in the Middle Wavelength Infrared. *Opt. Lett.* **31**, 616-618 (2006).

115. Devore, J.R. Refractive Indices of Rutile and Sphalerite. *J. Opt. Soc. Am.* **40**, 266-266 (1950).
